# Synergistic potentiation of antibiotics by chamomile phytochemicals against multidrug-resistant *Helicobacter pylori*

**DOI:** 10.1186/s13099-025-00777-2

**Published:** 2025-12-15

**Authors:** Menna M. M. Mohammed Ali, Hala Mohamed Abu Shady, Sayed. M. M, Hayam A. E. Sayed

**Affiliations:** 1 Department of Microbiology, Faculty of Science, Ain Shams University, Cairo, 11566 Egypt; 2 Department of internal medicine and gastroenterology, Faculty of medicine, Ain Shams University, Cairo, 11566 Egypt

**Keywords:** *Helicobacter pylori*- chamomile- antibiotic resistance -Molecular docking

## Abstract

**Background:**

*Helicobacter pylori* is a significant global health issue, infecting nearly half of the population. Increasing antibiotic resistance leads to treatment failures. This study examined the antibacterial effects of chamomile ethanolic extract, both alone and combined with standard antibiotics, as a potential approach to fighting antibiotic-resistant *H. pylori* strains.

**Methods:**

Thirty antral gastric biopsies were collected from patients undergoing diagnostic upper endoscopy at El-Demerdash Hospital in Cairo, Egypt. *H. pylori* isolates were identified by rapid urease test (RUT), cultured, and tested for antimicrobial susceptibility to clarithromycin (CLR, 15 µg), metronidazole (MET, 5 µg), amoxicillin (AX, 25 µg), tetracycline (TE, 30 µg), rifampicin (RA, 30 µg), and levofloxacin (LEV, 5 µg). The minimum inhibitory concentration (MIC) and minimum bactericidal concentration (MBC) were determined. The antibacterial activity of chamomile ethanolic extract was tested alone and in combination with these antibiotics. Phytochemical profiling was conducted using FT-IR and GC-MS. The identified compounds were analyzed through molecular docking against ten *H. pylori* targets: lipoprotein 20 (LPP20, HP1456), aspartate α-decarboxylase (ADC), S-ribosylhomocysteinase (LuxS), GTP cyclohydrolase II (GCH II), cytotoxin-associated gene A (CagA), sialic acid-binding adhesin (SabA), blood group antigen-binding adhesin (BabA), vacuolating cytotoxin A (VacA), and fructose-1,6-bisphosphate aldolase (FBA), using AutoDock Vina 1.5.7.

**Results:**

All thirty biopsies tested positive for RUT, although only 20 yielded successful cultures. The chamomile ethanolic extract demonstrated anti-*H. pylori* activity against all 20 isolates, with MIC values ranging from 1.562 to 6.25 mg/mL and MBC values from 3.12 to 12.5 mg/mL. When combined with antibiotics, the extract altered their antibacterial activity, primarily producing synergistic effects, while a few combinations exhibited antagonistic effects. Notably, in cases where antibiotics alone had limited activity, adding chamomile extract significantly enhanced effectiveness, increasing inhibition zones by 2.2-fold for tetracycline, 2.1-fold for rifampicin, and 1.7–2.3-fold for levofloxacin. FT-IR analysis confirmed the chemical safety of the extract, while GC-MS profiling identified 38 constituents, including 14 compounds with known antimicrobial properties. Molecular docking revealed strong binding affinities of eight bioactive compounds toward *H. pylori* target proteins. Additionally, Lipinski’s Rule of Five and ADMET profiling indicated these compounds possess favorable drug-like properties, including safety and oral bioavailability.

**Conclusions:**

Chamomile ethanolic extract shows promising anti-*H. pylori* activity and can enhance the effectiveness of standard antibiotics, indicating its potential as a complementary treatment to combat antibiotic-resistant *H. pylori* infections.

**Supplementary Information:**

The online version contains supplementary material available at 10.1186/s13099-025-00777-2.

## Background

*Helicobacter pylori (H. pylori*) is a widespread infection and a major public health concern, affecting nearly half of the world’s population, about 4.4 billion people. It mainly causes gastric infections, beginning with mild non-atrophic gastritis, which can progress to peptic ulcers, atrophic gastritis, gastric adenocarcinoma, and rarely, gastric MALT lymphoma. *H. pylori* infection can also impact other parts of the gastrointestinal tract, leading to duodenal ulcers and colorectal cancer [1, 2].

Currently, two main therapies are used to treat *H. pylori* infections. “Triple therapy” combines two antibiotics (metronidazole, clarithromycin, levofloxacin, or amoxicillin) with a proton pump inhibitor (PPI) [3], while “quadruple therapy” adds bismuth salts to the antibiotic regimen [4, 5]. Unfortunately, the rise of antibiotic-resistant *H. pylori* strains has led to increased treatment failure rates worldwide [6, 7]. This has sparked growing interest in medicinal plant extracts as alternative or supplementary therapies to help eradicate *H. pylori* [8, 9].

Phytotherapy, also known as herbal medicine, involves using plants or plant extracts to prevent or treat various diseases. Herbal remedies have been used to improve *H. pylori* eradication rates, manage gastrointestinal issues, and reduce antibiotic resistance and side effects [3]. Among these, *Matricaria chamomile*, a popular medicinal flowering plant in the Asteraceae family, has gained interest for its activity against multidrug-resistant bacteria. Chamomile grows either as an annual or perennial plant [10, 11] and is included in the pharmacopeias of 26 countries worldwide. Its flower heads are commonly used for therapeutic purposes [12]. Chamomile extracts have shown antibacterial activity against *Staphylococcus aureus*,* Pseudomonas aeruginosa*,* Listeria monocytogenes*,* Enterococcus faecalis*,* Escherichia coli*,* Acinetobacter baumannii*, and *Klebsiella pneumoniae* [13].

Chamomile was chosen for this study due to its well-known antimicrobial, anti-cancer, anti-inflammatory, and antioxidant properties [14–17]. The current research aimed to evaluate the anti-*H. pylori* activity of ethanolic chamomile extract, examine its synergistic effects with common antibiotics, and conduct in silico analyses to identify potential *H. pylori* cellular targets of the bioactive molecules found in the phytochemical analysis of the extract.

## Methods

### *H. pylori* isolation and antibiogram generation

#### Handling of gastric biopsy samples for *Helicobacter pylori* isolation

Antral biopsies that tested positive by rapid urease test (RUT) were collected from patients (18 males, 12 females; mean age, 43 ± 13 years) undergoing diagnostic upper endoscopy at El-Demerdash Hospital in Cairo, Egypt, between August 2023 and February 2024. Patients who had received antibiotics, proton pump inhibitors (PPIs), or bismuth salts within the previous 14 days were excluded. Each biopsy was placed in 1 mL of sterile phosphate-buffered saline (PBS; 0.1 M, pH 7.4) and immediately transported on ice to the Microbiology Department at Ain Shams University, Faculty of Science, for culturing.

#### Isolation and identification of *H. pylori*

Within one hour of collection, biopsies were homogenized in PBS using a sterile tissue grinder (mortar and pestle) and streaked onto Columbia blood agar (Oxoid, UK) supplemented with 7% sheep blood and antibiotic supplements (trimethoprim 5 mg/L, cefsulodin 5 mg/L, vancomycin 10 mg/L, and amphotericin B 5 mg/L). Plates were incubated at 37 °C under microaerophilic conditions (5% O₂, 10% CO₂; Campygen, Oxoid) for 3–10 days. After incubation, the isolates were mainly identified as *H. pylori* based on characteristic colony morphology (small, round, translucent colonies), positive urease and catalase activity, and Gram staining (Gram-negative, spiral-shaped rods). PCR detection was also performed for further analysis.

#### Antibiotic sensitivity test

The *H. pylori* isolates were tested against six antibiotics from different classes commonly used in treatment protocols, as shown in Table 1. Antibiotic susceptibility testing was performed using the disc diffusion method, following the guidelines of the Clinical and Laboratory Standards Institute (CLSI) (2020) [18]. Plates were incubated under microaerophilic conditions at 37 °C for 24–48 h. Zones of inhibition were measured in millimeters, and the results were interpreted based on the CLSI zone diameter breakpoints [18].

**Table 1:** Represent the antibiotics employed in the study

Antibiotic	Code	Class	Concentration (µg/disk)
Clarithromycin	CLR	Macrolides	15
Metronidazole	MET	Nitroimidazoles	5
Amoxicillin	AX	β lactams	25
Tetracycline	TE	Tetracyclines	30
Rifampin	RA	Ansamycins	30
Levofloxacin	LEV	Quinolones	5

## Molecular identification of *H. pylori*

### DNA extraction

Genomic DNA was extracted using the QIAamp DNA Mini Kit (Qiagen, Germany) following the manufacturer’s instructions. Briefly, 200 µL of ATL buffer and 20 µL of proteinase K were added to 200 µL of the sample to lyse it, and then the mixture was incubated for 10 min at 56 °C. To assist the binding of DNA to the QIAamp Mini Spin Column, 200 µL of ethanol (96%) was added. After vortexing for 15 s, the mixture was transferred to the QIAamp Mini spin column (in a 2 mL collection tube) and centrifuged at 6000 x g (8000 rpm) for 1 min to allow the DNA to adhere to the silica membrane within the column. Subsequently, three consecutive washing steps were performed using different buffers provided in the kit to purify the genomic DNA, according to the manufacturer’s instructions. Finally, after the purification, the eluted DNA was used as the template for the PCR reaction.

### Detection of ***H. pylori glmM*** gene and 16 S rRNA-specific gene

For the molecular detection of *H. pylori*, two specific genes were targeted: the *glmM* gene and the 16 S rRNA gene. The *glmM* gene is a housekeeping gene present in all *H. pylori* strains. It encodes phosphoglucosamine mutase, an enzyme that converts glucosamine-6-phosphate into glucosamine-1-phosphate, a precursor in the biosynthesis of N-acetylglucosamine. This pathway is essential for peptidoglycan synthesis, which maintains bacterial cell wall integrity and supports growth [19]. Conventional PCR assays were performed at the Animal Health Research Institute, Dokki, Giza, Egypt. Both genes were amplified in identical reaction mixtures with a final volume of 25 µL, including 5 µL of extracted DNA, 12.5 µL of EmeraldAmp GT PCR Master Mix (Takara), and 20 pmol of each primer. Primers for the *glmM* gene were adopted from Arfaee et al. [20], and primers for the 16 S rRNA gene from Ho et al. [21]. The sequences of the forward (F) and reverse (R) primers are listed in Table 2, and the PCR cycling conditions are summarized in Table 3. The expected amplicon sizes were 296 bp for *glmM* and 109 bp for 16 S rRNA. PCR products were analyzed on 2% agarose gels stained with ethidium bromide, using a 100 bp ladder as the molecular size marker. Each assay included both positive and negative controls. Electrophoresis was run for 30 min, after which the gels were visualized under UV light and photographed with a gel documentation system.

**Table 2:** Showing sequences of the primers used in different PCR reactions

Target gene	Primer sequences (5′−3′)	Target product (bp)	References
***H. pylori glmM***	F: GGA TAA GCT TTT AGG GGT GTT AGG GG	296	[20]
	R: GCT TAC TTT CTA ACA CTA ACG CGC		
***H. pylori-specific***16 S rRNA gene	F: CTG GAG AGA CTA AGC CCT CC	109	[21]
	R: ATT ACT GAC GCT GAT TGT GC		

**Table 3:** Showing different PCR conditions used in the study

H. pylori PCR conditions	
**glmM gene**	***H. pylori*** **specifc-16srRNA**
An initial denaturation at 94 °C for 5 min	An initial denaturation at 94 °C for 5 min
35 cycles of denaturation at 94º C for 30 s	35 cycles of denaturation at 94º C for 30 s
Annealing at 57º C for 40 s	Annealing at 50º C for 30 s
Extension 72˚C for 40 s	Extension 72˚C 30 s
Final extension at 72º C for 10 min	Final extension 72˚C 7 min

### Preparation of plant extract

Dried chamomile flowers (*Matricaria chamomilla*) were purchased from local markets in Cairo, Egypt, for testing against *H. pylori*. Ethanolic extract was prepared using the soaking method with a 1:10 (w/v) plant-to-solvent ratio. Specifically, 10 g of dried flowers were soaked in 100 mL of 99.8% ethanol for 48 h in a sterilized flask sealed with a cork [22]. After soaking, the mixture was filtered through Whatman No. 1 filter paper, and the filtrate was sterilized by passing through a 0.22 μm Millipore filter. The final extract concentration was adjusted to 100 mg/mL.

#### Screening of anti-*H*. *Pylori* activity for chamomile extract by the disc diffusion method

According to CLSI guidelines [18], the disc diffusion method was used to evaluate the activity of chamomile extract against pure *H. pylori* isolates. Bacterial suspensions were adjusted to 1.5 × 10⁸ CFU/mL and inoculated onto Mueller–Hinton agar (Himedia, India) supplemented with 7% sheep blood. Sterile blank discs (6 mm in diameter) were loaded with 20 µL of the extract, allowed to diffuse for one hour, and then incubated at 37 °C under microaerophilic conditions for 24–48 h. Zones of inhibition were measured in millimeters. All experiments were performed in triplicate, and the average diameter of the inhibition zones was recorded.

#### Screening of the synergistic effect of chamomile extract with antibiotics using the disc diffusion method

All *H. pylori* isolates were inoculated onto Mueller–Hinton agar plates supplemented with 7% defibrinated sheep blood. Bacterial suspensions were adjusted to 1.5 × 10⁸ CFU/mL (0.5 McFarland standard) and evenly spread over the agar surface using a sterile cotton swab. Six antibiotic discs, as listed in the antibiotic susceptibility tests in Table 1, were placed on each plate, and 20 µL of chamomile extract (100 mg/mL) was carefully applied to each disc. Plates were pre-diffused for 1 h at 4 °C, then incubated at 37 °C for 24 h under microaerophilic conditions. Inhibition zone diameters were measured and compared with those of the antibiotics alone and the chamomile extract alone. Combination effects were classified as synergistic if the inhibition zone of the combination exceeded the sum of the zones of the individual agents, additive if the combination zone equaled the sum, and antagonistic if the combination zone was smaller than the sum of the individual zones [23, 24].

#### Determination of the minimum inhibitory concentration and minimum bactericidal concentration for chamomile ethanolic extract

The minimum inhibitory concentration (MIC) of chamomile extract against *H. pylori* was determined using the microbroth dilution method according to CLSI (2020), with Mueller–Hinton broth (Himedia, India) supplemented with lysed sheep blood as the medium. A stock solution of 100 mg/mL extract was serially diluted two-fold in a sterile 96-well microtiter plate to obtain concentrations ranging from 50 mg/mL to 0.39 mg/mL, with 100 µL per well. Fresh bacterial cultures were adjusted to 0.5 McFarland turbidity in sterile 0.85% NaCl, and 10 µL was added to each well. Plates were incubated at 37 °C under microaerophilic conditions for 24–48 h, and the MIC was defined as the lowest concentration showing no visible bacterial growth. Positive (inoculum only) and negative (extract only) controls were included. The minimum bactericidal concentration (MBC) was determined by subculturing 100 µL from wells showing visible growth inhibition in the MIC assay onto Mueller–Hinton agar supplemented with 7% sheep blood, followed by incubation under microaerophilic conditions at 37 °C for 24–48 h; the lowest concentration preventing colony formation was recorded as the MBC [22].

### Tolerance level

The tolerance level of chamomile extract was determined using the ratio of MBC to MIC (MBC/MIC). This ratio was used to classify the antimicrobial activity of the extract as either bacteriostatic or bactericidal. Extracts with an MBC/MIC ratio ≥ 4 were considered bacteriostatic, while those with an MBC/MIC ratio < 4 were considered bactericidal. This method provides a straightforward way to evaluate whether the extract inhibits growth or kills bacterial cells [25].

### Statistical analysis

A one-way ANOVA was performed to statistically analyze the data using SPSS software, version 23. When a difference was *p* < 0.05, it was regarded as statistically significant.

## Phytochemical analysis of the chamomile extract

Phytochemical analysis of the chamomile extract was performed using FTIR to confirm the absence of CN⁻ groups for safety reasons and to identify the functional groups present. Additionally, the extract’s chemical components were identified and quantified through GC–MS analysis.

### FTIR analysis

Fourier Transform Infrared Spectroscopy (FTIR) was used to identify the functional groups in the chamomile extract. This technique detects infrared light absorption at specific wavelengths that correspond to characteristic vibrations of chemical bonds, producing a spectrum with peaks indicating particular functional groups. The analysis was performed at the Central Laboratory, Faculty of Science, Ain Shams University, Cairo, Egypt.

### GC – MS analysis

Before gas chromatography–mass spectrometry (GC–MS) analysis, the chamomile extract was dried and derivatized by suspending it in 50 µL of bis(trimethylsilyl) trifluoroacetamide (BSTFA) with 1% trimethylchlorosilane (TMCS) and 50 µL of pyridine to convert functional groups into trimethylsilyl (TMS) derivatives. GC–MS analysis was performed using an Agilent Technologies system, consisting of a 7890B gas chromatograph coupled with a 5977 A mass selective detector, at the Central Laboratories Network of the National Research Centre, Cairo, Egypt. A DB-5MS column (30 m × 0.25 mm internal diameter, 0.25 μm film thickness) was used. The temperature program included an initial 60 °C for 1 min, followed by a ramp to 320 °C at 10 °C/min, with a 10-minute hold. A splitless injection of 1 µL was used, with hydrogen as the carrier gas at a flow rate of 1.0 mL/min. The injector and detector temperatures were maintained at 300 °C and 320 °C, respectively. Mass spectra were recorded by electron ionization (EI) at 70 eV over the m/z range of 50–800, with a solvent delay of 6 min, a quadrupole temperature of 150 °C, and a mass transfer line temperature of 230 °C. Compounds were identified by comparing fragmentation patterns with those in the Wiley and NIST Mass Spectral Libraries.

## In-silico investigation of the potential targets within *H. pylori*

Based on previous studies [26–36], ten potential protein targets involved in the survival, colonization, and pathogenesis of *H. pylori* were selected for an in-silico study to analyze the interactions of each bioactive compound found in chamomile extract using AutoDock Vina 1.5.7. The chosen targets and their respective control ligands are listed in Table 4.

**Table 4:** The different targets within *H. pylori* and their control ligands were used in the study

Target name	PDB ID	Its function	The control ligand name	Reference for target
Urease	1E9Y	-Bacterial Survival -Virulence factor	Acetohydroxamic Acid	[37]
Lipoprotein 20 (LPP20; HP 1456)	5OK8	-colonization -pathogenesis	Mitomycin	[38]
Aspartate α-decarboxylase (ADC) enzyme	1UHE	-survival -maintain intracellular pH	Aspartate	[39]
S-ribosylhomocysteinase (LuxS)	1J6X	Works as a signaling molecule crucial for bacterial communication.	-	[40]
GTP cyclohydrolase II (GCH II)	4RL4	-survival and colonization	-	[41]
Cytotoxin-associated gene A (CagA)	4dvz	-virulence factor	-	[42]
Sialic acid-binding adhesion (SabA)	4o5j	-adhesion -pathogenesis	-	[43]
Blood group antigen-binding adhesion (BabA)	4zh7	-adhesion -pathogenesis	Lewis b antigen	[44]
Vacuolating cytotoxin A (VacA)	2qv3	-virulence factor	-	[45]
fructose-1,6-bisphosphate aldolase (FBA)	3C56	-Energy production for survival	3-(hydroxy[(phosphonooxy) acetyl] amino} propyl dihydrogen phosphate (PH4)	[46]

### Target selection and preparation

The three-dimensional (3D) structures of *H. pylori* protein targets were obtained in PDB format from the RCSB Protein Data Bank (http://www.rcsb.org/pdb/home/home.do) [47]. The proteins listed in Table 4 were prepared by removing water molecules, adding hydrogen atoms and Kollman charges, and checking for and correcting any missing residues. Subsequently, the protein structures were converted to PDBQT format using AutoDock Vina 1.5.7 for further molecular docking studies.

### Ligand preparation

After GC–MS analysis of chamomile extract, compounds present at higher concentrations and known for their antibacterial activity were selected as ligands for docking studies against H. pylori targets. Ligand preparation involved retrieving the 3D structures of these compounds from the PubChem database (https://pubchem.ncbi.nlm.nih.gov/) [48] in Structure Data File (SDF) format. Geometry optimization and energy minimization were then performed using the Auto Optimization tool, followed by the Merck Molecular Force Field 94 (MMFF94) in Avogadro 1.2.0, to generate stable, low-energy conformations. The optimized ligand structures were subsequently converted to Protein Data Bank (PDB) format for use in molecular docking studies.

### Molecular docking study and visualization of protein-ligand interaction

Molecular docking was performed using AutoDock Vina 1.5.7, which requires both the receptors and ligands to be in PDBQT format. Protein binding sites were identified by visualizing interactions with control ligands and based on information from previous studies involving these proteins. For each target, a grid box was created to cover the active site and facilitate ligand binding. Grid center coordinates and box dimensions for each target are listed in Table 5. The binding energy (kcal/mol) between ligand and protein served as the main parameter for docking. Ligand–protein interactions were visualized with Discovery Studio (DS) Visualizer software 2021 to examine how compounds bind to specific amino acid residues within the target proteins.

**Table 5:** The grid center points and box dimensions for each target

Target name	Grid center points			Grid box dimensions		
	X	Y	Z	X	Y	Z
Urease	129.663	126.647	88.689	40	40	40
LPP20 (HP 1456)	37.505	44.426	48.01	40	42	40
ADC	−11.001	83.078	13.762	66	72	50
LUXS Protein	−15.243	14.230	8.398	56	54	56
GCH II	−0.904	20.509	−12.599	60	46	48
CagA protein	−4.857	38.96	−17.797	40	40	40
SabA protein	87.111	−35.344	17.147	40	40	40
BabA protein	−34.051	−20.947	49.014	52	46	48
VacA protein	9.811	49.175	−8.77	78	62	48
FBA	−2.499	0.715	−9.888	40	64	48

### Drug-likeness and pharmacokinetic profiling

The pharmacokinetic profiles of the selected compounds were assessed by first applying Lipinski’s rule of five to evaluate drug-likeness, followed by in-silico ADMET (Absorption, Distribution, Metabolism, Excretion, and Toxicity) predictions. Compounds with favorable docking scores and interactions were then prioritized for further pharmacokinetic analysis.

#### Lipinski’s rule of five

Lipinski’s rule of five is a well-established method for assessing the drug-likeness of compounds and their potential for oral activity. These rules evaluate the physical and chemical properties of a compound, along with its similarity to existing drugs, to predict pharmacological effects and biological activity. According to this rule, an orally active compound should not violate more than one of the following criteria: a molecular weight under 500 Da, a logP value below 5, fewer than five hydrogen bond donors, and fewer than ten hydrogen bond acceptors [49].

#### In silico ADMET prediction

A drug is a molecule that can reach a specific target site and remain in the body long enough to produce a biological response. To evaluate these properties, ADMET analysis was performed. The selected compounds were assessed for absorption, distribution, metabolism, excretion, and toxicity using web-based prediction tools, including SwissADME (http://www.swissadme.ch/index.php) [50], pkCSM (https://biosig.lab.uq.edu.au/pkcsm/) [51], and ProTox-II (https://toxnew.charite.de/protox_II/index.php? site=compound_input) [52]. The canonical SMILES of all molecules were obtained from the PubChem database and uploaded to the ADMET prediction servers for analysis.

## Results

### Laboratory isolation and identification of ***H. pylori***: positive cultures in 20/30 biopsies

**Fig. 1:**
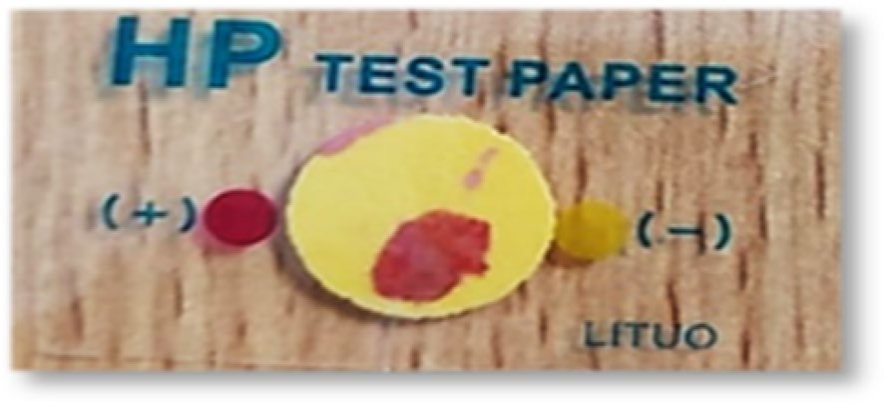
Positive rapid urease test for gastric biopsy

**Fig. 2:**
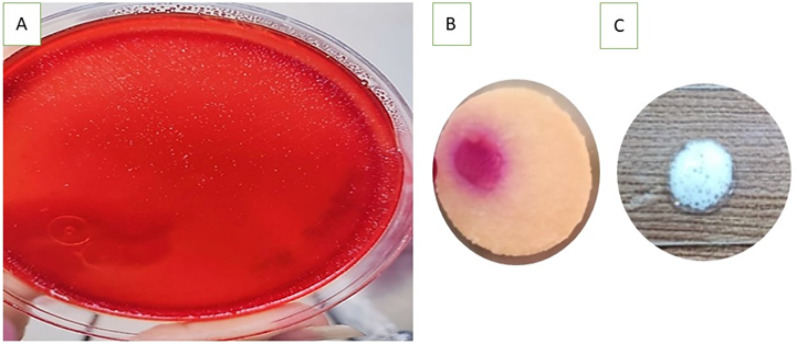
Primary identification of *H. pylori* isolates; **A**: small and transparent colonies on Columbia Blood Agar; **B**: positive rapid urease test; **C**: positive catalase test

Although all thirty samples tested positive for RUT, only twenty produced positive cultures. The colonies obtained were characteristic of *H. pylori*, as they were small, transparent, and tested positive for urease and catalase, as shown in Figs. 1 and 2. Under microscopic examination, the cells appeared in the typical form of *H. pylori*, which is a gram-negative spiral or curved rod.

### Antibiotic resistance and grouping of ***H. pylori*** isolates

**Fig. 3:**
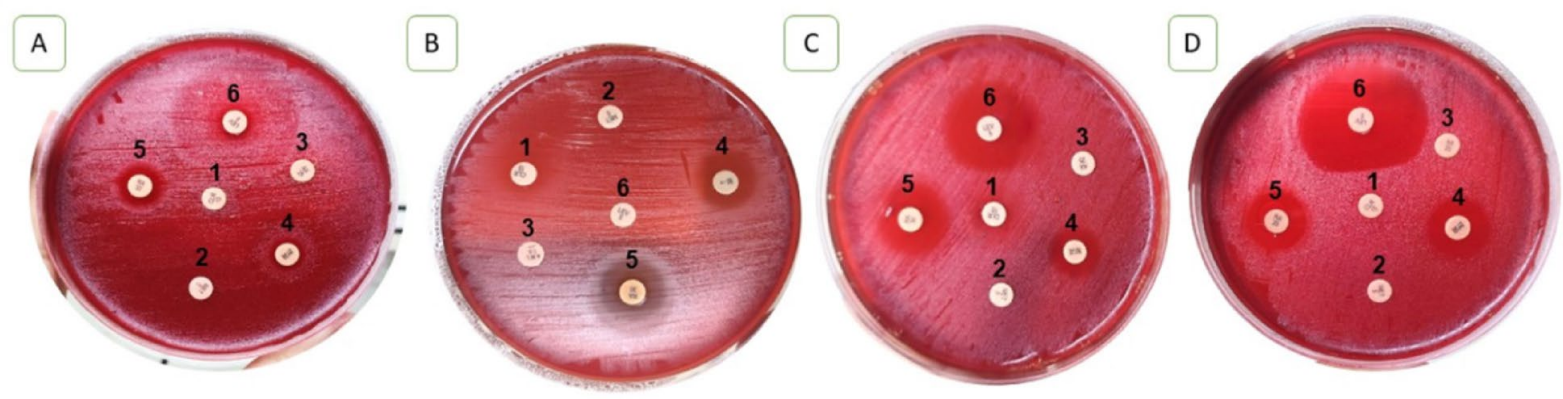
The antibiotic sensitivity representative plates for each group of *H. pylori* isolates; each symbol from A to D represents a different group, (**A**) representative plate for group 1 of *H. pylori* isolates; (**B**) representative plate for group 2 of *H. pylori* isolates; (**C**) representative plate for group 3 of *H. pylori* isolates; (**D**) representative plate for group 4 of *H. pylori* isolates. Each number from (1–6) represents an antibiotic,1 = CLR (15 µg); 2 = MET (5 µg); 3 = AX (25 µg); 4 = TE (30 µg); 5 = RA (30 µg); and 6 = LEV (5 µg)

**Fig. 4:**
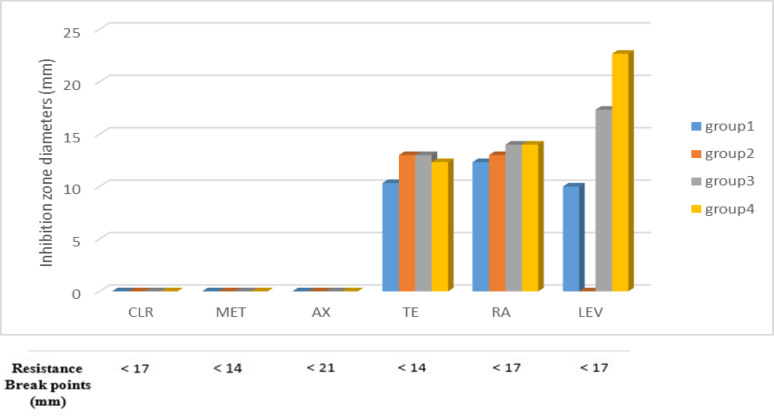
Histogram represents the antibiotic sensitivity of the tested *H. pylori* isolates against its therapeutic antibiotics; MET (5 µg): metronidazole; AX (25 µg): amoxicillin; CLR (15 µg): clarithromycin; TE (30 µg): tetracycline; RA (30 µg): rifampicin; LEV (5 µg): levofloxacin

Since neither EUCAST nor CLSI guidelines specify disc diffusion breakpoints for *H. pylori*, we relied on previous studies to interpret our results. Resistance was defined as an inhibition zone diameter of less than 21 mm for clarithromycin, less than 17 mm for metronidazole, rifampicin, and levofloxacin, and less than 14 mm for amoxicillin and tetracycline [53]– [54]. Based on these criteria, all isolates were resistant to clarithromycin, metronidazole, amoxicillin, tetracycline, and rifampicin, while 60% remained susceptible to levofloxacin, as shown in **(**Figs. 3 and 4; Tables 6 and 7**)**. The isolates were grouped into four groups based on antibiotic sensitivity results: Group 1 (four isolates) showed inhibition zones of 10.33 ± 0.33 mm (TE, 30 µg), 12.33 ± 0.33 mm (RA, 30 µg), and 10 ± 0.00 mm (LEV, 5 µg), with no inhibition for other antibiotics; Group 2 (four isolates) exhibited 13 ± 0.00 mm (TE, 30 µg) and 13 ± 0.00 mm (RA, 30 µg), with no inhibition for LEV (5 µg), CLR (15 µg), MET (5 µg), or AX (25 µg); Group 3 (four isolates) demonstrated inhibition zones of 13 ± 0.00 mm (TE, 30 µg), 14 ± 0.00 mm (RA, 30 µg), and 17.33 ± 0.33 mm (LEV, 5 µg); and Group 4 (eight isolates) showed inhibition zones of 12.33 ± 0.33 mm (TE, 30 µg), 14 ± 0.00 mm (RA, 30 µg), and 22.66 ± 0.33 mm (LEV, 5 µg), as shown in Fig. 4.

**Table 6:** Egyptian *H. pylori* antibiogram in El-Demerdash hospital

Antibiotic	Percentage susceptibility (%)
Metronidazole (5 µg)	**0**
Amoxicillin (25 µg)	**0**
Clarithromycin (15 µg)	**0**
Levofloxacin (5 µg)	**60%**
Tetracycline (30 µg)	**0**
Rifampicin (30 µg)	**0**

Bold values represent the susceptibility percentages against tested antibiotics.

**Table 7:** The Inhibition zone diameter of antibiotics and chamomile extract in (mm) against all *H. pylori* isolates

Isolate number	Inhibition zone diameter in mm (± SE)						
	CLR (15 µg)	MET (5 µg)	AX (25 µg)	TE (30 µg)	RA (30 µg)	LEV (5 µg)	Cham extract
1	0.00 ± 0.00	0.00 ± 0.00	0.00 ± 0.00	10.33 ± 0.33	12.33 ± 0.33	10 ± 0.00	24.33 ± 0.33
2	0.00 ± 0.00	0.00 ± 0.00	0.00 ± 0.00	13 ± 0.00	13 ± 0.00	0.00 ± 0.00	14 ± 0.00
3	0.00 ± 0.00	0.00 ± 0.00	0.00 ± 0.00	12.33 ± 0.33	14 ± 0.00	22.66 ± 0.33	15 ± 0.00
4	0.00 ± 0.00	0.00 ± 0.00	0.00 ± 0.00	10.33 ± 0.33	12.33 ± 0.33	10 ± 0.00	24.33 ± 0.33
5	0.00 ± 0.00	0.00 ± 0.00	0.00 ± 0.00	12.33 ± 0.33	14 ± 0.00	22.66 ± 0.33	15 ± 0.00
6	0.00 ± 0.00	0.00 ± 0.00	0.00 ± 0.00	10.33 ± 0.33	12.33 ± 0.33	10 ± 0.00	24.33 ± 0.33
7	0.00 ± 0.00	0.00 ± 0.00	0.00 ± 0.00	13 ± 0.00	13 ± 0.00	0.00 ± 0.00	14 ± 0.00
8	0.00 ± 0.00	0.00 ± 0.00	0.00 ± 0.00	12.33 ± 0.33	14 ± 0.00	22.66 ± 0.33	15 ± 0.00
9	0.00 ± 0.00	0.00 ± 0.00	0.00 ± 0.00	10.33 ± 0.33	12.33 ± 0.33	10 ± 0.00	24.33 ± 0.33
10	0.00 ± 0.00	0.00 ± 0.00	0.00 ± 0.00	13 ± 0.00	13 ± 0.00	0.00 ± 0.00	14 ± 0.00
11	0.00 ± 0.00	0.00 ± 0.00	0.00 ± 0.00	13 ± 0.00	14 ± 0.00	17.33 ± 0.33	15.33 ± 0.33
12	0.00 ± 0.00	0.00 ± 0.00	0.00 ± 0.00	12.33 ± 0.33	14 ± 0.00	22.66 ± 0.33	15 ± 0.00
13	0.00 ± 0.00	0.00 ± 0.00	0.00 ± 0.00	13 ± 0.00	14 ± 0.00	17.33 ± 0.33	15.33 ± 0.33
14	0.00 ± 0.00	0.00 ± 0.00	0.00 ± 0.00	12.33 ± 0.33	14 ± 0.00	22.66 ± 0.33	15 ± 0.00
15	0.00 ± 0.00	0.00 ± 0.00	0.00 ± 0.00	13 ± 0.00	13 ± 0.00	0.00 ± 0.00	14 ± 0.00
16	0.00 ± 0.00	0.00 ± 0.00	0.00 ± 0.00	13 ± 0.00	14 ± 0.00	17.33 ± 0.33	15.33 ± 0.33
17	0.00 ± 0.00	0.00 ± 0.00	0.00 ± 0.00	12.33 ± 0.33	14 ± 0.00	22.66 ± 0.33	15 ± 0.00
18	0.00 ± 0.00	0.00 ± 0.00	0.00 ± 0.00	12.33 ± 0.33	14 ± 0.00	22.66 ± 0.33	15 ± 0.00
19	0.00 ± 0.00	0.00 ± 0.00	0.00 ± 0.00	13 ± 0.00	14 ± 0.00	17.33 ± 0.33	15.33 ± 0.33
20	0.00 ± 0.00	0.00 ± 0.00	0.00 ± 0.00	12.33 ± 0.33	14 ± 0.00	22.66 ± 0.33	15 ± 0.00
Mean	0.00 ± 0.00	0.00 ± 0.00	0.00 ± 0.00	12.19 ± 0.22	13.46 ± 0.15	18.16 ± 1.25	16.73 ± 0.83
P-value	-	-	-	0.000	0.000	0.000	0.000

### PCR detection of ***H. pylori*** genes: 1 of 4 isolates positive for ***glmM***

**Fig. 5:**
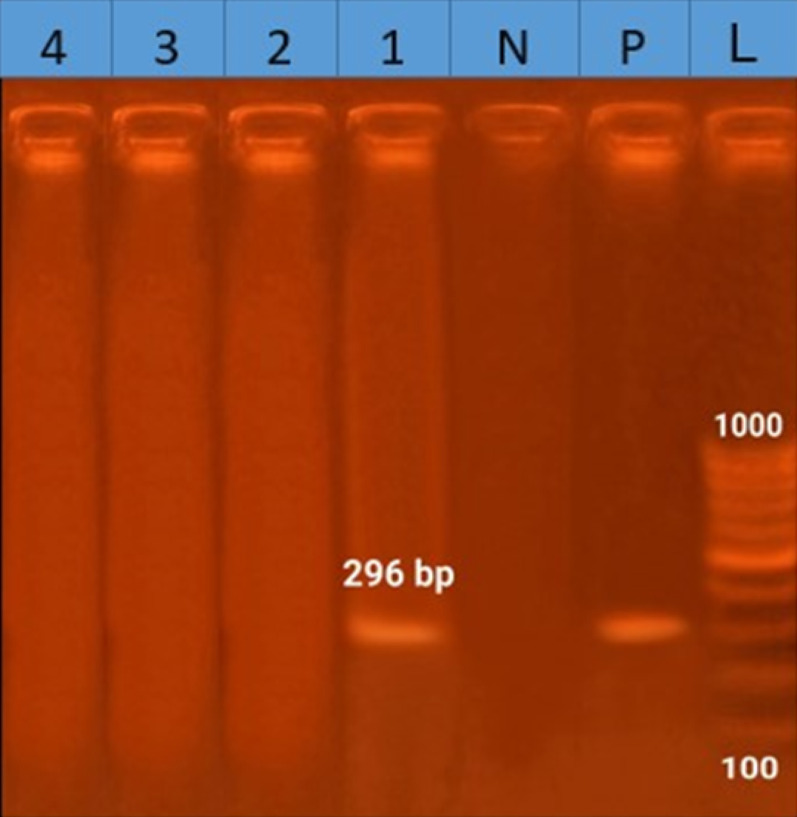
Agarose gel electrophoresis of PCR products from *H. pylori* isolates targeting specific genes. The 296 bp fragment was only detected in sample 1. L: molecular weight marker (100 bp ladder). Lane P: positive control. Lane N: negative control. Lanes 1, 2, 3, 1,2,3,4 are *H. pylori* isolates

In this study, conducted at a single hospital in Egypt, *H. pylori* isolates were grouped based on their antibiotic sensitivity profiles. One isolate from each group was randomly chosen for PCR detection of the *H. pylori*-specific *glmM* and 16 S rRNA genes. Four isolates with typical colony morphology and positive results in both RUT and catalase assay were analyzed. Only the isolate from group 1 produced the expected 296 bp *glmM* band, as shown in Fig. 5. The other three isolates were negative for *glmM*, and all four tested negative for the *H. pylori*-specific 16 S rRNA gene.

### Dual interactions between chamomile extract and antibiotics: synergism and antagonism

**Fig. 6:**
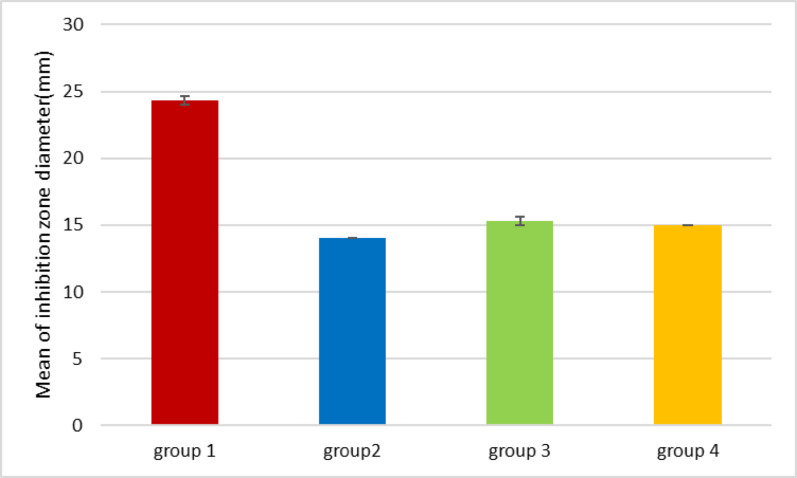
Shows the mean inhibition zone diameters of H. pylori isolates against chamomile ethanolic extract in (mm). Error bars represent the standard error of the mean (SEM). *P* < 0.05

The tested plant extract showed anti-*H. pylori* activity, with an average inhibition zone diameter of 16.73 ± 0.87 mm. The inhibition zones for groups 1–4 were 24.33 ± 0.33, 14.00 ± 0.00, 15.33 ± 0.33, and 15.00 ± 0.00 mm, respectively, as shown in Fig. 6 and Table 7. When combined with antibiotics, chamomile extract exhibited varying effects depending on the *H. pylori* isolate, revealing two types of synergism. In groups 2 and 4, chamomile extract alone produced zones of 14.00 ± 0.00 mm and 15.00 ± 0.00 mm, while (CLR, 15 µg) and (MET, 5 µg) alone have no inhibition; however, when combined with chamomile extract, inhibition zones increased to 16.33 ± 0.33 mm and 16.00 ± 0.00 mm in group 2 respectively, and 15.33 ± 0.33 mm for both antibiotics in group 4, indicating **antibiotic synergistic to chamomile effect**. **Mutual synergism** was also observed: (TE, 30 µg) and (RA, 30 µg) in group 2 increased from 13.00 ± 0.00 mm alone to 28.66 ± 0.88 mm (2.2-fold) and 27.33 ± 0.33 mm (2.1-fold), respectively, when combined with chamomile extract. In group 4, (TE, 30 µg), (RA, 30 µg), and (LEV, 5 µg) alone produced zones of 12.33 ± 0.33 mm, 14.00 ± 0.00 mm, and 22.66 mm, which increased to 28.66 ± 0.33 mm (2.3-fold), 29.33 ± 0.33 mm (2.1-fold), and 38.33 ± 0.88 mm (1.7-fold), respectively, when combined with chamomile extract. For isolates in group 3, LEV (5 µg) alone produced a zone of 17.33 ± 0.33 mm, which increased to 34.33 ± 0.33 mm (1.9-fold) with the addition of chamomile extract, highlighting the enhanced activity of both agents.

Additionally, two distinct types of antagonism were observed between chamomile extract and antibiotics. In the first type, **antibiotics antagonistic to chamomile** (CLR, 15 µg; MET, 5 µg; AX, 25 µg) showed no inhibitory effect when tested alone, whereas the chamomile extract demonstrated strong inhibition. When combined, the inhibition zones were smaller than those of chamomile alone, indicating reduced sensitivity of *H. pylori*. For example, in group 1, chamomile alone produced a zone of 24.33 ± 0.33 mm, while combinations with clarithromycin (CLR, 15 µg), amoxicillin (AX, 25 µg), and metronidazole (MET, 5 µg) resulted in zones measuring 13.33 ± 0.33 mm, 11.33 ± 0.33 mm, and 11 ± 0.00 mm, respectively. In group 3, chamomile alone produced a zone of 15.33 ± 0.33 mm, while the combination treatments yielded zones of 14.33 ± 0.33 mm (CLR, 15 µg) and 14 ± 0.00 mm (AX, 25 µg; MET, 5 µg), which were again smaller than the zones for chamomile alone.

**Fig. 7:**
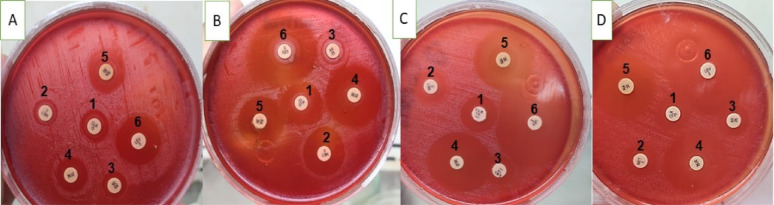
The combined action of chamomile extract and the antibiotics against *H. pylori* isolates different groups, where: (**A**) showing group 1 representative plate; (**B**) showing group 2 representative plate; (**C**) showing group 3 representative plate; and (**D**) showing group 4 representative plate. Each number from (1–6) represents a tested antibiotic,1 = clarithromycin (CLR, 15 µg); 2 = metronidazole (MET, 5 µg); 3 = amoxicillin (AX, 25 µg); 4 = tetracycline (TE, 30 µg); 5 = rifampicin (RA, 30 µg); 6 = levofloxacin (LEV, 5 µg)

**Fig. 8:**
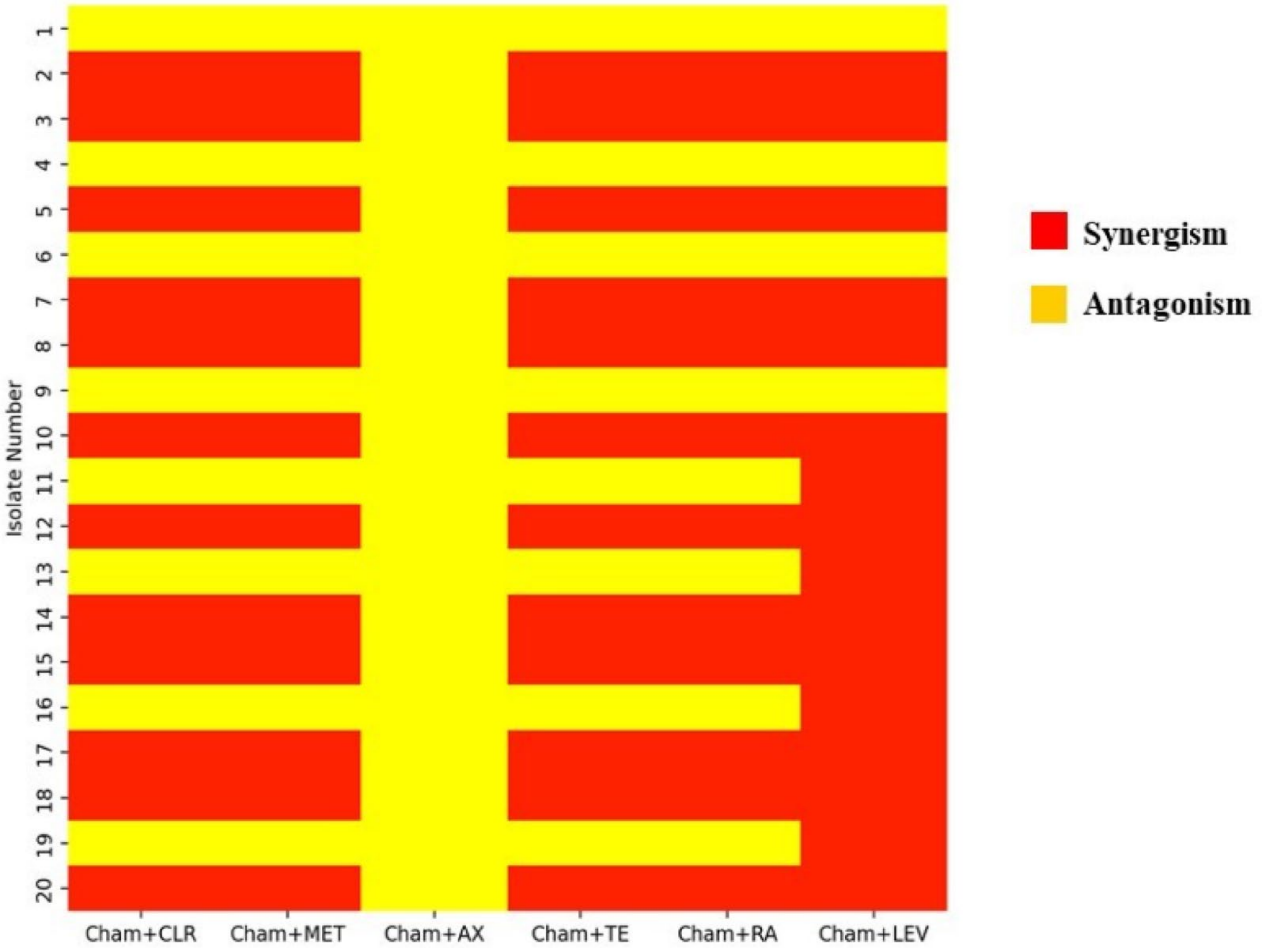
The heat map shows the effectiveness of the combined action of chamomile ethanolic extract with antibiotics based on inhibition zone diameter values shown in Table 8

The second type, mutual antagonism, was observed with tetracycline (TE, 30 µg) and rifampicin (RA, 30 µg), where both the antibiotic and chamomile extract individually inhibited *H. pylori*. However, the combined zones were smaller than the sum of their individual effects. For example, in Group 1, TE, RA, and levofloxacin (LEV, 5 µg) alone produced diameters of 10.33 ± 0.33 mm, 12.33 ± 0.33 mm, and 10.00 ± 0.00 mm, respectively. When combined with chamomile, the inhibition zones measured 14.66 ± 0.33 mm, 15 ± 0.00 mm, and 16.66 ± 0.33 mm for TE, RA, and LEV, respectively. Similarly, in Group 3, TE and RA alone produced 13 ± 0.00 mm and 14 ± 0.00 mm, respectively, resulting in zones of 24.33 ± 0.66 mm (TE, 30 µg) and 22 ± 0.00 mm (RA, 30 µg) with chamomile, still less than the sum of their individual effects, confirming mutual antagonism. Synergistic and antagonistic interactions of chamomile with antibiotics are represented in Table 8; Figs. 7 and 8. The effect of chamomile and its combination with antibiotics was statistically significant (*P* < 0.05).

**Table 8:** Mean of Inhibition zone diameter of the combination between chamomile extract and antibiotics against all *H. pylori* isolates

isolate number	Cham + CLR	Cham + MET	Cham +AX	Cham +TE	Cham +RA	Cham +LEV
1	13.33 ± 0.33	11.33 ± 0.33	11 ± 0.00	14.66 ± 0.33	15 ± 0.00	16.66 ± 0.33
2	16.33 ± 0.33	16 ± 0.00	13 ± 0.00	28.66 ± 0.88	27.33 ± 0.33	33.33 ± 0.33
3	15.33 ± 0.33	15.33 ± 0.33	12.66 ± 0.33	28.66 ± 0.33	29.33 ± 0.33	38.33 ± 0.88
4	13.33 ± 0.33	11.33 ± 0.33	11 ± 0.00	14.66 ± 0.33	15 ± 0.00	16.66 ± 0.33
5	15.33 ± 0.33	15.33 ± 0.33	12.66 ± 0.33	28.66 ± 0.33	29.33 ± 0.33	38.33 ± 0.88
6	13.33 ± 0.33	11.33 ± 0.33	11 ± 0.00	14.66 ± 0.33	15 ± 0.00	16.66 ± 0.33
7	16.33 ± 0.33	16 ± 0.00	13 ± 0.00	28.66 ± 0.88	27.33 ± 0.33	33.33 ± 0.33
8	15.33 ± 0.33	15.33 ± 0.33	12.66 ± 0.33	28.66 ± 0.33	29.33 ± 0.33	38.33 ± 0.88
9	13.33 ± 0.33	11.33 ± 0.33	11 ± 0.00	14.66 ± 0.33	15 ± 0.00	16.66 ± 0.33
10	16.33 ± 0.33	16 ± 0.00	13 ± 0.00	28.66 ± 0.88	27.33 ± 0.33	33.33 ± 0.33
11	14.33 ± 0.33	14 ± 0.00	14 ± 0.00	24.33 ± 0.66	22 ± 0.00	34.33 ± 0.33
12	15.33 ± 0.33	15.33 ± 0.33	12.66 ± 0.33	28.66 ± 0.33	29.33 ± 0.33	38.33 ± 0.88
13	14.33 ± 0.33	14 ± 0.00	14 ± 0.00	24.33 ± 0.66	22 ± 0.00	34.33 ± 0.33
14	15.33 ± 0.33	15.33 ± 0.33	12.66 ± 0.33	28.66 ± 0.33	29.33 ± 0.33	38.33 ± 0.88
15	16.33 ± 0.33	16 ± 0.00	13 ± 0.00	28.66 ± 0.88	27.33 ± 0.33	33.33 ± 0.33
16	14.33 ± 0.33	14 ± 0.00	14 ± 0.00	24.33 ± 0.66	22 ± 0.00	34.33 ± 0.33
17	15.33 ± 0.33	15.33 ± 0.33	12.66 ± 0.33	28.66 ± 0.33	29.33 ± 0.33	38.33 ± 0.88
18	15.33 ± 0.33	15.33 ± 0.33	12.66 ± 0.33	28.66 ± 0.33	29.33 ± 0.33	38.33 ± 0.88
19	14.33 ± 0.33	14 ± 0.00	14 ± 0.00	24.33 ± 0.66	22 ± 0.00	34.33 ± 0.33
20	15.33 ± 0.33	15.33 ± 0.33	12.66 ± 0.33	28.66 ± 0.33	29.33 ± 0.33	38.33 ± 0.88
Mean	14.93 ± 0.22	14.39 ± 0.36	12.66 ± 0.21	24.99 ± 1.18	24.59 ± 1.19	32.19 ± 1.75
P-value	0.000	0.000	0.000	0.000	0.000	0.000

### Chamomile extract inhibits and kills ***H. pylori***: MIC and MBC determination

The ethanolic extract of chamomile inhibited *H. pylori*, with MICs varying among isolate groups, indicating differences in susceptibility (range: 1.56–6.25 mg/mL). The lowest MIC (1.56 mg/mL) was found in the second group of isolates (four isolates), while the highest MIC (6.25 mg/mL) was observed in the third group (four isolates). Corresponding MBCs ranged from 3.12 to 12.5 mg/mL, with groups 1, 2, and 4 showing MBCs of 3.12 mg/mL, and group 3 having an MBC of 12.5 mg/mL **(**Supplementary Table S1**)**. The MBC/MIC ratios, shown in Table 9, indicate that chamomile extract displayed bactericidal activity against all *H. pylori* groups at concentrations close to their MICs.

**Table 9:** Represent MIC and MBC of chamomile extract against *H. pylori* groups

H. pylori groups	Chamomile extract		
	MIC	MBC	MBC/MIC
GP1	3.12	3.12	1
GP2	1.56	3.12	2
GP3	6.25	12.5	2
GP4	3.12	3.12	1

### Phytochemical analysis of chamomile extract: FT-IR and GC-MS characterization

**Fig. 9:**
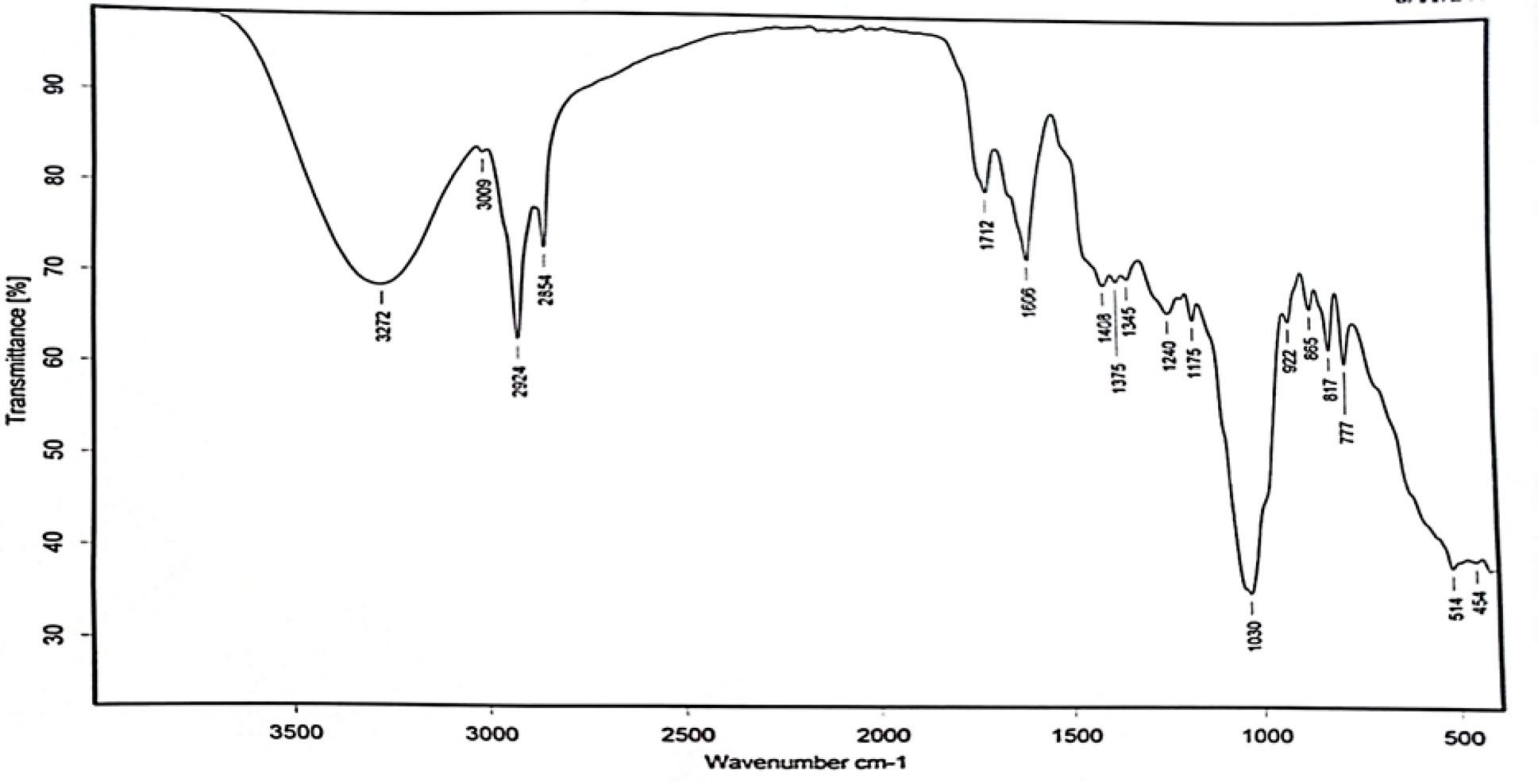
FT-IR analysis of *Matricaria chamomilla* (chamomile) ethanolic extract

The FT-IR spectrum of chamomile ethanolic extract, as shown in Fig. 9, reveals various functional groups. A peak at 3272 cm⁻¹ is typically linked to the stretching vibration of hydrogen-bonded hydroxyl groups (-OH), while the peak at 2924 cm⁻¹ corresponds to the C–H stretching vibrations of alkanes. Another peak at 2854 cm⁻¹ is also related to C–H stretching, specifically the symmetric stretching of CH₂ groups in alkanes. The peak at 1712 cm⁻¹ is commonly attributed to the carbonyl (C = O) stretching vibration of ketones, aldehydes, or esters, whereas the peak at 1606 cm⁻¹ indicates the C = C stretching vibration of aromatic rings. Additionally, the peak at 1030 cm⁻¹ is often associated with C–O stretching vibrations in functional groups such as phenols and ethers. Notably, hazardous groups like cyano (C ≡ N: 2220–2260 cm⁻¹) and acetylenic (C ≡ C: 2100–2200 cm⁻¹) were absent, suggesting that chamomile ethanolic extract is safe, consistent with previous studies using FT-IR for safety assessment [55].

**Fig. 10:**
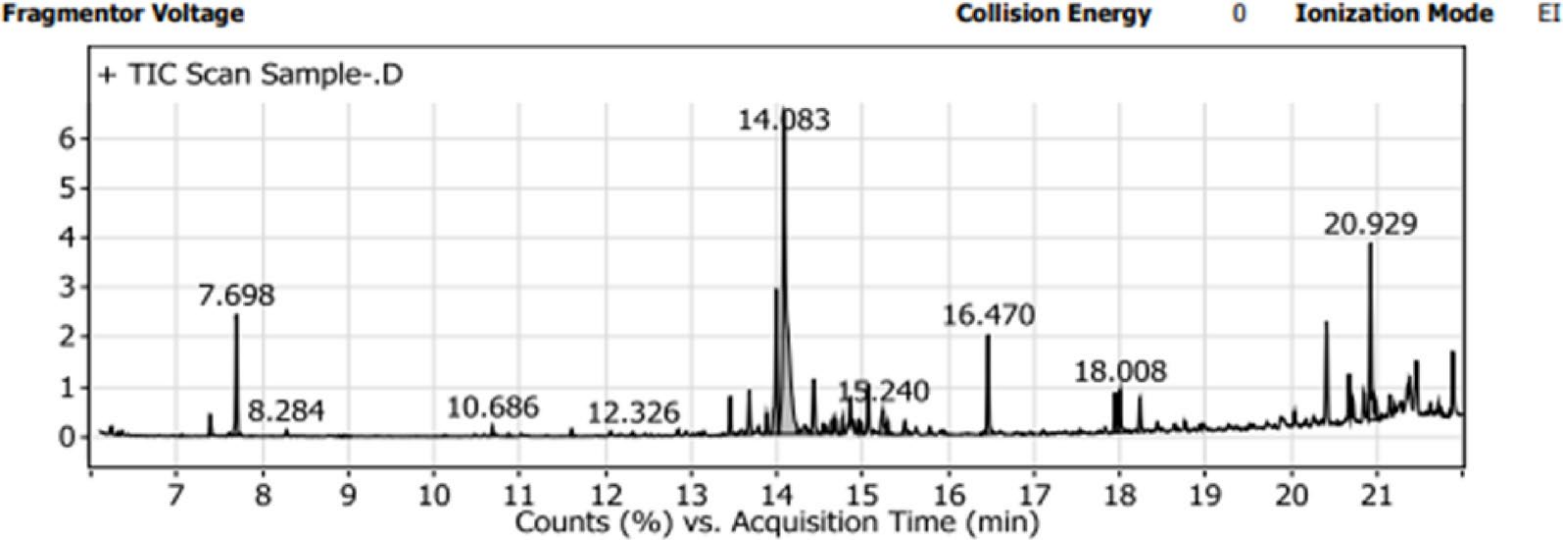
Chromatogram of chamomile ethanolic extract from GC-MS analysis

GC-MS analysis identified thirty-eight compounds in the chamomile extract, as shown in Fig. 10, including saturated and unsaturated fatty acids, carboxylic acids, sugar alcohols, glycosides, amino acids, and sugars. Among these, fourteen compounds such as palmitic acid, oleic acid, 9,12-octadecadienoic acid, stearic acid, benzoic acid, xylonic acid, myo-inositol, myristic acid, erythritol, butanedioic acid, heptanoic acid, octanoic acid, and nonanoic acid have been previously reported to possess antibacterial activity, enhance the activity of other compounds, or exhibit synergistic effects with antibiotics, as shown in Table 10.

**Table 10:** The chemical components of chamomile possess antimicrobial activity

compound name	Retention time (RT)	Area%	Its effect	Ref.
Palmitic Acid, TMS derivative	16.47	5.4	Antibacterial	[55, 56]
Oleic Acid, (Z)-, TMS derivative	18.008	2.76	Antibacterial	[58]
9,12-Octadecadienoic acid (Z, Z)-, TMS derivative	17.949	2.19	Antibacterial	[60]
Stearic acid, TMS derivative	18.242	1.94	Antibacterial	[55–57]
Xylonic acid, 2,3,4-tris-O-(trimethylsilyl)-, delta. -lactone, D-	13.461	1.85	Antibacterial and improve the antimicrobial activity of other compounds	[59]
Benzoic Acid, TMS derivative	7.391	1.12	Antibacterial	[60]
cis-11,14-Eicosadienoic acid, tert-butyldimethylsilyl ester	13.885	1.06	Antibacterial	[55]
Myo-Inositol, 6TMS	15.496	1.06	Antibacterial and improve the antimicrobial activity of other compounds	[61]
Myristic acid, TMS derivative	14.544	0.53	Antibacterial	[62]
Erythritol, 4TMS derivative	10.686	0.45	Antibacterial	[63–66]
Butanedioic acid, 2TMS derivative	8.284	0.27	Antibacterial	[66]
Heptanoic acid, TMS derivative	6.322	0.2	Antibacterial	[55]
Octanoic acid, TMS derivative	7.61	0.12	Antibacterial	[67]
Nonanoic acid, TMS derivative	8.899	0.11	Antibacterial	[68, 69]

### Computational study identifies chamomile bioactive compounds with strong ligand-protein interactions in ***H. pylori***

**Fig. 11:**
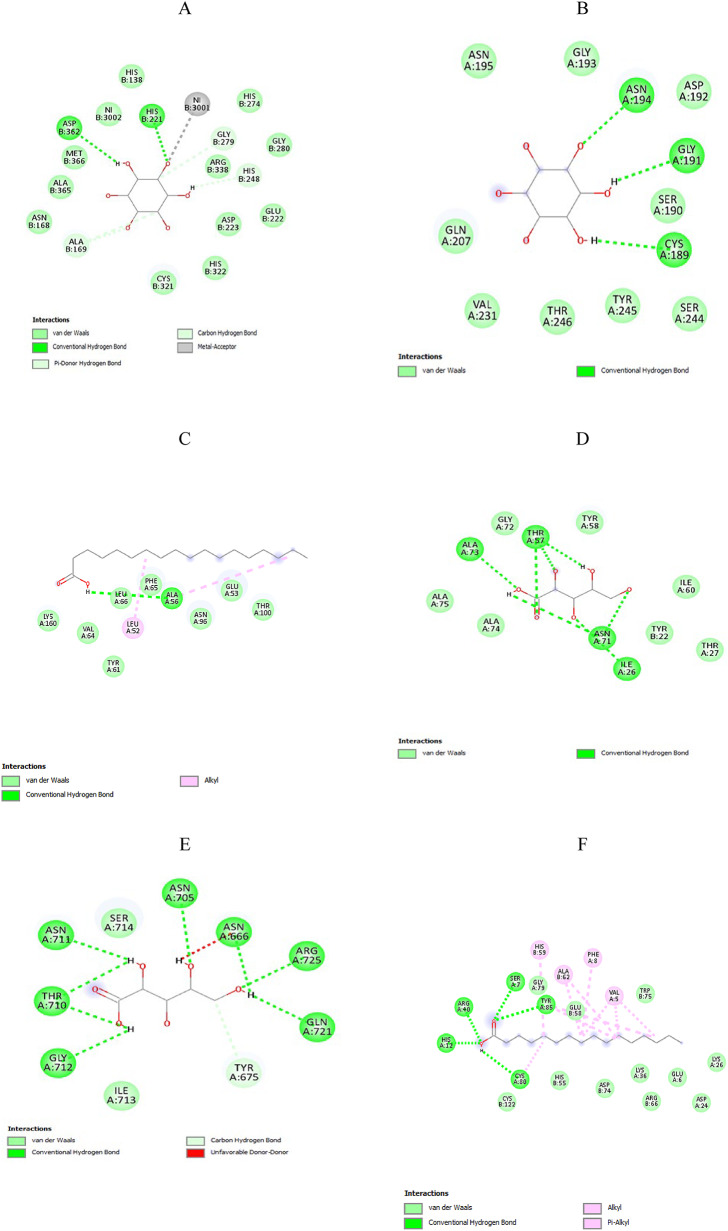

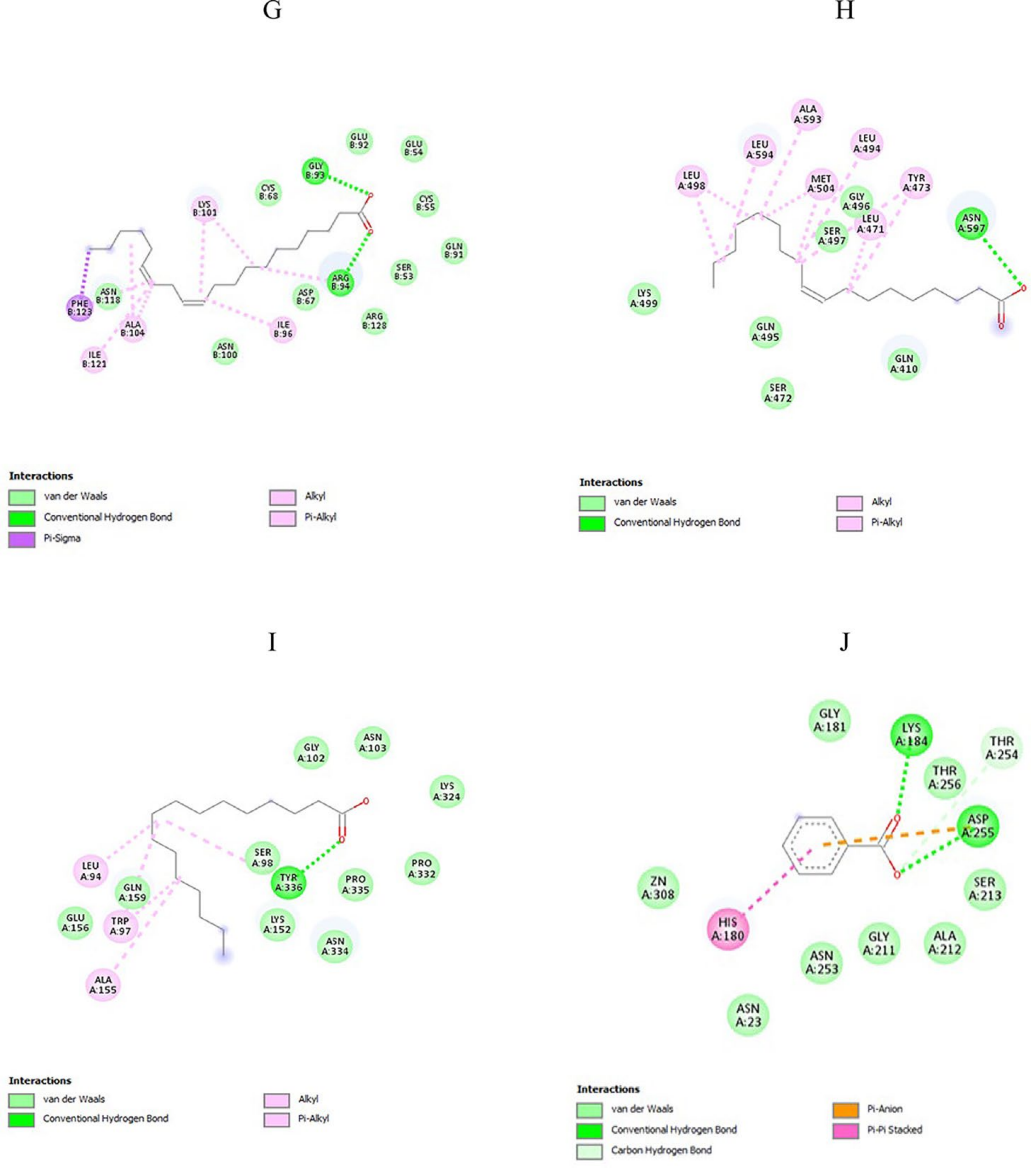
Shows 2D representations of interactions between ligands and active site residues of the target protein. (**A**) Depicts the interaction of myo-inositol with urease; (**B**) depicts the interaction of myo-inositol with BabA protein; (**C**) depicts the interaction of stearic acid with LPP20 (HP 1456); (**D**) depicts the interaction of xylonic acid with ADC enzyme; (**E**) depicts the interaction of xylonic acid with VacA protein; (**F**) depicts the interaction of palmitic acid with LuxS protein; (**G**) depicts the interaction of eicosadienoic acid with GCH II enzyme; (**H**) depicts the interaction of oleic acid with CagA protein; (**I**) depicts the interaction of octadecadienoic acid with SabA protein; (**J**) depicts the interaction of benzoic acid with FBA enzyme

An in silico study showed that chamomile extract can significantly impact *H. pylori* by targeting various essential cellular components, especially enzymes. Of the fourteen antibacterial compounds found in the extract, eight demonstrated high binding affinities with specific *H. pylori* targets compared to control ligands. Lower binding energy values represent more stable ligand–protein complexes, as summarized in Table 11, while detailed interactions between ligands and target proteins are illustrated in Table 12; Fig. 11. These promising compounds include myo-inositol, xylonic acid, stearic acid, palmitic acid, cis-11,14-eicosadienoic acid, oleic acid, octadecadienoic acid, and benzoic acid. Interactions between control ligands and target proteins are shown in Supplementary Table S2 and Fig. S1.

**Table 11:** The binding affinities (kcal/mol) of chamomile bioactive compounds to *H. pylori* targets

Tested ligand	Target within H. pylori	Its binding affinity(kcal/mol)	Control ligand	Its binding affinity (kcal/mol)
Myo-inositol	Urease	−6.1	Acetohydroxamic acid	−4.4
	BabA protein	−6.4	Lewis b antigen	−5.9
Stearic acid	LPP20 (HP 1456)	−6.3	Mitomycin (control drug)	−6.1
Xylonic acid	ADC	−4.8	Aspartate	−3.9
	VacA protein	−6.7	-	-
Palmitic acid	LuxS Protein	−6.4	-	-
Eicosadienoic acid	GCH II	−6.4	-	-
Oleic acid	CagA protein	−6.0	-	-
Octadecadienoic acid	SabA protein	−5.8	-	-
Benzoic acid	fructose-1,6-bisphosphate aldolase	−7.0	3-(hydroxy[(phosphonooxy) acetyl] amino} propyl dihydrogen phosphate (PH4)	−6.3

**Table 12:** Represent the difRepresent the different interactions between tested ligands and target proteins of *H. pylori*

Tested Ligand	Target within H. pylori	Type of interactions	Bond length
Myo-inositol	Urease	−2 Hydrogen bonds ASP B:362, His B:221 -Metal-acceptor interaction with NI B3001 -Carbon -hydrogen interaction with GLY B: 279, AlA B:169 -π-donor hydrogen bond: His B: 248 van der Waals interactions with ARG B:338, HIS A:274, GLY A: 280, GLU B:222, ASP B: 223, HIS B: 322, CYS B: 321, ASN B: 168, ALA B: 365, MET B: 366, HIS B: 138 and NI B: 3002	-For H-bond: (1.92–2.98 Å) For metal-acceptor: 2.82 Å -For C-H bond: (3.28–3.6Å) -For π-donor hydrogen bond: 2.88 Å
	BabA protein	-Hydrogen bond with ASN A: 194, GLY A:191 and CYS A: 189 -van der Waals interactions with ASN A: 195, GLY A: 193, ASP A: 192, SER A: 190, GLN A: 207, VAL A:231, SER A: 244, TYR A: 245, THR A: 246	-For H-bond: (2.23–2.60Å)
Stearic acid	LPP20 (HP 1456)	-Hydrogen bond with ALA A:56 -Alkyl interactions with LEU A:52 and ALA A:56 -van der Waals interactions with LYS A: 160, VAL A: 64, LEU A:66, PHE A:65, ASN A:96, GLU A:53, THR A: 100	-For H-bond: 2.76 Å - For alkyl interaction: (4.28, 4.87 Å)
Xylonic acid	ADC	-Hydrogen bond each with ILE A:26, ALA A:73, two hydrogen bonds with ASN A:71and three hydrogen bond with THR A:57 -van der Waals interactions ILE A:60, THR A: 27, ALA A:74, ALA A:75, GLY A:72, TYR A:58 and TYR B:22	-For H-bond: (2–2.82.82Å)
	VacA protein	-Hydrogen bond each with ASN A: 705, GLN A: 721 and two hydrogen bonds with ASN A:666, ASN A:710, ASN A: 711, GLY A: 712, ARG A: 725 -Carbon hydrogen bond with TYR A:675 van der Waals interactions with TYR A:675, ILE A:713 and SER A: 714 Unfavorable interaction with ASN A: 666	For H-bond: (2.10–2.80 Å) For the C-H bond: (3.74 Å) For unfavorable interaction: 1.62 Å
Palmitic acid	LuxS Protein	-Hydrogen bond each with TRP A:85, ARG A:40, HIS A:12, SER A:7 and CYS A:80 -Alkyl interactions: VAL A:5, PHE A:8, HIS B:59 and ALA B:62 Pi- alkyl interaction with ALA B: 62, HIS B:59, TYR A:85 and PHE A:8 van der Waals interactions with GLY A:79, GLU B:58, TRP B:75, GLU A:6, ARG B:66, LYS A:36, ASP B:74, HIS B:55 and CYS B:122	-For H-bond: (2.43–2.76 Å) -For alkyl interaction: (4–5.25.25Å) For pi-alkyl interaction: (4.90–5.25 Å)
Eicosadienoic acid	GCH II	-Two hydrogen bonds with GLY B:93 and ARG B:94 -Alkyl interaction: ILE B:121, ALA B:104, LYS B:101and ILE B: 96 -π-Alkyl interaction and π-sigma interaction: PHE B:123 Van der waals interaction with CYS B:68, GLU B:92, GLU B:54, CYS B:55, GLN B:91, SER B:53, ARG B:128, ASP B:67, ASN B: 100 and ASN B:118	-H-bond: (2.04 Å) - For alkyl interaction: (4.21–5.42 Å) -π-Alkyl interaction: 5 Å -π-sigma interaction: 3.89 Å
Oleic acid	CagA protein	-Hydrogen bond with ASN A: 597 -Alkyl interaction: LEU A:471, TYR A:473, LEU A:494, LEU A: 498, MET A:504, ALA A:593 and LEU A:594 Pi- alkyl interaction with TYR A: 473 Van der waals interaction with GLY A:496, SER A:497, GLN A:410, SER A:472, GLN A:495 and LYS A: 499	-For H-bond: 2.24 Å -For alkyl interaction: (3.73–5.47 Å) -Pi- alkyl interaction: 4.97 Å
9,12-Octadecadienoic acid	SabA protein	-Hydrogen bond with TYR A:336 -Alkyl interaction: LEU A:94, TRP A:97, ALA A:155 and TYR A:336 -Pi-alkyl interaction with TYR A:97 and TYR A:336 -Van der Waals interaction with GLY A: 102, ASN A:103, LYS A: 324, PRO A: 332, PRO A: 335, ASN A:334, LYS A: 152, SER A:98, GLU A: 156, and GLN A: 159	-For H-bond: 1.91 Å -For alkyl interaction: (4.40–5.50Å) -Pi-alkyl interaction: (4.58–4.90 Å)
Benzoic acid	FBA	-Two hydrogen bonds with LYS A: 184 and ASP A:255 -Carbon hydrogen bond: THR A:254 -π-π stacked interaction: HIS A:180 π-anion interaction: ASPA:255 van der Waals interaction with GLY A: 181, THR A:256, SER A: 213, ALA A: 212, GLY A: 211, ASN A:253, ASN A: 23, and Zn A: 308	-For H-bond: (2.15–2.83 Å) -For C-H bond: 3.68 Å -π-π stacked interaction:4.57Å -π-anion interaction:4.47 Å

### In Silico ADMET and Lipinski analysis indicate favorable drug-like properties of chamomile compounds

The eight compounds from chamomile extract that showed strong binding affinities with various *H. pylori* targets were further evaluated for their ADMET properties, as shown in Table 13. Lipinski’s rule of five was also used to assess the drug-likeness of each compound, ensuring their potential safety and effectiveness. All eight compounds exhibited either no violations or only one violation of Lipinski’s rules, as demonstrated in Table 14.

**Table 13:** Predicted Pharmacokinetics of the eight tested compounds of chamomile extract

Parameter	Myo-inositol	Stearic acid	Xylonic acid	Octadecadienoic acid	Eicosadienoic acid	Oleic acid	Palmitic acid		Benzoic acid	
GIA	Low	High	Low	High	High	High	High	High		
BBB	No	No	No	No	No	No	Yes	Yes		
P-gP substrate	No	No	No	No	No	No	No	No		
CYP1A2 inhibitor	No	No	No	No	No	No	No	No		
CYP2C19 inhibitor	No	No	No	No	No	No	No	No		
CYP2C9 inhibitor	No	No	No	No	No	No	No	No		
CYP2D6 inhibitor	No	No	No	No	No	No	No	No		
CYP3A4 inhibitor	No	No	No	No	No	No	No	No		
PAINS	No	No	No	No	No	No	No	No		

**Table 14:** Lipinski rule of five for the selected eight compounds in the chamomile extract

Ligand name	Molecular Weight	Hydrogen bond acceptor	Hydrogen bond donor	LogP	Lipinski rule	
					Meet RO5	Violation
Palmitic acid	256.42	2	1	6.732	Yes	1
Oleic acid	282.260	2	1	6.169	Yes	1
Octadecadienoic acid	314.250	4	2	5.8	Yes	1
Stearic acid	284.27	2	1	7.571	Yes	1
Xylonic acid	166.050	6	5	−2.016	Yes	0
Benzoic acid	122.12	2	1	1.957	Yes	0
Eicosadienoic acid	342.280	4	2	6.801	Yes	1
Myo-inositol	180.060	6	6	−3.057	Yes	1

GIA (gastrointestinal absorption). BBB (Blood Brain Barrier). PgP (P-glycoprotein transporter). CYP1A2, CYP2C19, CYP2C9, CYP2D6 and CYP3A4 are the five types of cytochromes P450 (CYP). PAINS (Pan Assay Interference).

## Discussion

*Helicobacter pylori* is a worldwide health issue, with a rising number of reports of antibiotic resistance, including among children. A 2024 meta-analysis by Salahi-Niri et al. [70]., who examined data from 28 countries across five WHO regions, emphasized the increasing problem of multidrug resistance in *H. pylori*. These results are consistent with our findings.

In this study, 30 gastric biopsies that tested positive with RUT were collected from patients at the hospital. Of these, 20 biopsies were successfully cultured on selective media, producing colonies with the characteristic morphology of *H. pylori*. The lower number of positive cultures compared to RUT results may be due to the uneven distribution of *H. pylori* in the gastric mucosa, loss of bacterial viability during transport, or the inability of some strains to grow on blood agar, either because they are deeply embedded in the mucosa or have transformed into the unculturable coccoid form [71]– [72].

The antibiotic susceptibility of these 20 isolates, obtained from Egyptian patients with gastric diseases, was tested against six antibiotics (clarithromycin 15 µg, amoxicillin 25 µg, metronidazole 5 µg, tetracycline 30 µg, rifampicin 30 µg, and levofloxacin 5 µg) using the disc diffusion method.

The results revealed a significant problem of antibiotic resistance. All isolates were completely resistant to five of the six antibiotics tested, based on breakpoints reported in previous studies. Only levofloxacin showed inhibitory activity, with 60% of isolates being susceptible. These findings are consistent with other studies. For example, El-Shouny et al. [73]. observed 100% resistance to amoxicillin, 95% to metronidazole, and 90% to clarithromycin. Similarly, another study reported resistance rates of 54.3% for amoxicillin, 73.9% for metronidazole, 47.8% for clarithromycin, and 4.3% for tetracycline [74]. Goudarzi et al. [75]. also noted resistance to amoxicillin (27.7%), metronidazole (73.8%), clarithromycin (43.1%), and levofloxacin (13.4%). Furthermore, Metwally et al. [53]. found 100% resistance to metronidazole and 95% to amoxicillin, along with 20% and 40% resistance to levofloxacin and clarithromycin, respectively.

However, contradictory results have also been reported. Sadeghi et al. [76]. observed high resistance rates (>15%) for levofloxacin and tetracycline, but low resistance (≤ 15%) to amoxicillin. Abu-Gharbia et al. [77]. reported that 91.8% of isolates were resistant to rifampicin, while only 9.2% showed resistance to amoxicillin. Conversely, Li et al. [78]. found that all isolates were susceptible to amoxicillin, but 60.9% were resistant to rifampicin. Together, these studies, along with our findings, highlight the variability in *H. pylori* antibiotic susceptibility both worldwide and within the same geographical region.

Based on the resistance patterns, the 20 isolates in our study were divided into four categories, with each group sharing the same antibiotic susceptibility profile. A representative isolate from each group was selected for PCR analysis. Genomic DNA was extracted under consistent conditions, and annealing temperatures were optimized for each primer set to enhance specificity and accuracy. Two species-specific genes were targeted. The *glmM* gene primers were used to evaluate sequence polymorphisms among the isolates. A 296-bp band was amplified only in isolate 1 of group 1, while no amplification occurred in the other three isolates, indicating genotypic variability alongside phenotypic resistance differences. This finding aligns with prior studies, such as those by Abid et al. [79]. and Alnaji et al. [80]., which reported the low sensitivity of *glmM*-based PCR due to sequence polymorphisms and strain-to-strain variation.

For the 16 S rRNA gene, Hp1/Hp2 primers were tested for their ability to detect *H. pylori* from pure colonies. However, no 109-bp amplicon was obtained from any of the four isolates. Consistent with previous reports by Chong et al. [81]. and Chisholm et al. [82]., these primers appear to lack specificity for *H. pylori*, as they can cross-react with human DNA or gastric microbiota from gastric biopsies, resulting in false-positive results. In our study, they instead produced false negatives, even with pure *H. pylori* colonies. Nonetheless, the isolates were confidently identified as *H. pylori* based on their morphology (Gram-negative curved rods), strong urease activity, and the presence of small, transparent colonies on selective media.

The above findings emphasize the urgent need for local drug susceptibility monitoring and cautious antibiotic use, as *H. pylori* resistance patterns vary significantly across regions. Due to this increasing resistance, the anti-*H. pylori* activity of chamomile extract was tested as a possible alternative or supplement to traditional antibiotics.

In light of this, the ethanolic extract of chamomile flowers was tested against 20 *H. pylori* isolates. It produced a mean inhibition zone diameter of 16.73 mm, with a mean MIC of 3.43 mg/mL and a mean MBC of 4.99 mg/mL. These findings agree with those of Malm et al. [22]., who reported strong activity of chamomile ethanolic extract against *H. pylori* ATCC 43,504, although with lower MIC values (31.3–62.5 µg/mL) and MBC values (125–250 µg/mL). Such differences reflect the variability in strain susceptibility to the same compound. Consistent with this, another study reported that chamomile extract killed 40.9% of 31 antibiotic-resistant *H. pylori* strains [83], supporting its potential role in fighting multidrug-resistant isolates. Furthermore, Zulfiqar [84] demonstrated that chamomile extract inhibits urease, a crucial enzyme essential for *H. pylori* survival and colonization.

To enhance the effectiveness of antibiotics and combat resistance, the synergistic effects of chamomile ethanolic extract when combined with antibiotics were investigated. Synergism occurs when the combined effect of an extract and an antibiotic exceeds the sum of their individual effects. Two types of synergistic interactions were identified in this study.

The first type involved antibiotics synergistic to chamomile extract. In groups 2 and 4 of *H. pylori* isolates (60% of the isolates), clarithromycin and metronidazole showed no inhibitory activity when used alone, while chamomile extract did produce inhibition. When combined with the extract, the inhibition zones were larger than those of the extract alone, indicating a synergistic effect.

The second type was mutual synergism, where both the antibiotic and the extract were individually inhibitory, and their combined effect was greater than the sum of their individual effects. This was observed with tetracycline and rifampicin in groups 2 and 4 (60% of isolates), and with levofloxacin in groups 2 and 3 (40% of isolates). Similar synergistic effects of chamomile extract with other antibiotics, such as vancomycin, metronidazole, and tetracycline, have been reported against *Staphylococcus aureus* and *Clostridioides difficile* [85].

However, antagonistic interactions were also observed in 40% of isolates (groups 1 and 3). The first type, antibiotic antagonistic to chamomile extract, occurred when the antibiotics alone showed no inhibitory effect but reduced the activity of the chamomile extract when combined (observed with clarithromycin, amoxicillin, and metronidazole). The second type, mutual antagonism, occurs when both the extract and antibiotic are individually inhibitory, but their combined effect is less than the sum of their individual effects (as observed with tetracycline and rifampicin). Similar antagonistic interactions have been reported with other plant extracts and antibiotics against other Gram-negative bacteria such as *E. coli* and *P. aeruginosa* (e.g., *Picralima nitida* extract combined with ciprofloxacin and norfloxacin) [86]. These results demonstrate that not all plant–antibiotic combinations improve antibacterial effectiveness, highlighting the importance of careful evaluation before therapeutic use.

Phytochemical analysis of the ethanolic chamomile extract was performed to determine its composition and safety. FT-IR analysis revealed that harmful groups such as cyano and acetylenic groups were absent, confirming the extract’s safety.

GC-MS analysis identified thirty-eight compounds in the extract. Of these, fourteen with the highest relative abundance have been previously reported to exhibit antibacterial activity. Additionally, two compounds, xylonic acid, 2,3,4-tris-O-(trimethylsilyl)-δ-lactone, D-, and myo-inositol, 6TMS, are known to boost the antibacterial activity of other compounds [55–69], highlighting the potential for synergistic effects in chamomile extract.

Our findings differ from those reported by **Móricz **et al. [87]., who attributed the antibacterial activity of chamomile ethanolic extract to cis- and trans-spiroethers, coumarins such as herniarin and umbelliferone, and noted the presence of only small amounts of fatty acids (oleic, palmitic, and stearic acids), which they reported as having no antibacterial activity. Similarly, Singh et al. [11]. identified α-bisabolol, chamazulene, trans-β-farnesene, α-farnesene, β-eudesmol, spathulenol, germacrene D, berkheyaradulene, and β-selinene sesquiterpene compounds as the active antibacterial components in chamomile flowers.

These discrepancies between our GC-MS results and those of previous studies can be attributed to multiple factors that influence the chemical composition of chamomile. Factors such as geographic origin, environmental conditions, plant cultivars, and genetic differences can affect metabolite profiles. Additionally, variations in drying techniques and extraction methods can significantly alter the abundance and types of bioactive compounds present [88].

To investigate the mode of action by which chamomile extract affects *H. pylori* isolates, a computational study was conducted. Molecular docking was conducted using two categories of *H. pylori* targets. The first category included survival-related proteins, such as urease, ADC, GCH II, LuxS, and FBA. Docking with these targets may help clarify the growth-inhibitory effects observed in the MIC assays. The second category consisted of virulence- and colonization-related proteins, including CagA, VacA, BabA, SabA, and Lpp20 (HP1456). Although these proteins do not directly affect bacterial survival, they are essential for adhesion, toxin-mediated damage, and inflammation, which contribute to the pathogenicity of *H. pylori*.

It is crucial to highlight the role of these targets in *H. pylori*. Urease is essential for bacterial survival in the acidic environment of the stomach. It promotes colonization of the gastric mucosa and hydrolyzes urea into ammonia (NH₃) and carbon dioxide (CO₂), two key factors in the development of ulcers [89].

The outer membrane protein Lpp20 activates tumor necrosis factor-alpha (TNF-α) and promotes epithelial-mesenchymal transition (EMT), a process that can trigger metastasis and support cancer progression. Lpp20 also enhances cell motility, decreases E-cadherin expression in gastric cancer cells, and stimulates cell proliferation [38].

 ADC catalyzes the α-decarboxylation of L-aspartate to β-alanine, which is essential for producing pantothenate (vitamin B5), a precursor of Coenzyme A (CoA), a cofactor vital for enzymatic activity in all living organisms. ADC is found in *H. pylori* and over 200 other pathogens, making it a promising broad-spectrum therapeutic target [90-91].

 LuxS has a dual role in *H. pylori*: it is involved in quorum sensing. Also, it plays a metabolic role in cysteine biosynthesis from homocysteine, which is essential for bacterial growth and survival. In our study, LuxS was chosen as a target because of its metabolic function [92]. GCH II catalyzes the conversion of GTP to 2,5-diamino-6-β-ribosyl-4(3 H)-pyrimidinone-5′-phosphate, a rate-limiting step in riboflavin biosynthesis crucial for *H. pylori* survival [41].

Adhesion proteins BabA and SabA are essential for attaching to host cell receptors [93-94]. The toxins VacA and CagA are key factors in bacterial pathogenicity [95]. Lastly, FBA plays a major role in glycolysis and gluconeogenesis, ensuring a steady supply of ATP and metabolic intermediates. Inhibiting this enzyme can greatly disrupt bacterial growth and survival within the host [46].

Molecular docking was conducted with fourteen chemical compounds identified in chamomile extract through GC-MS analysis. Among these, eight compounds, including myo-inositol, stearic acid, xylonic acid, palmitic acid, cis-11,14-eicosadienoic acid, oleic acid, 9,12-octadecadienoic acid, and benzoic acid, showed the highest binding affinities compared to their respective control ligands.

Myo-inositol showed the strongest binding with urease and BabA, with binding energies of − 6.1 kcal/mol and − 6.4 kcal/mol, respectively. Xylonic acid exhibited binding energies of − 4.8 kcal/mol with ADC and − 6.7 kcal/mol with VacA. The other compounds also displayed high affinities for different targets: stearic acid with Lpp20 (–6.3 kcal/mol), palmitic acid with LuxS (–6.4 kcal/mol), cis-11,14-eicosadienoic acid with GCH II (–6.4 kcal/mol), oleic acid with CagA (–6.0 kcal/mol), 9,12-octadecadienoic acid with SabA (–5.8 kcal/mol), and benzoic acid with FBA (–7.0 kcal/mol).

The docking results also showed the high stability of the complexes, as demonstrated by relatively short bond lengths and the presence of multiple types of interactions that support their stability. These findings suggest that chamomile extract may not only serve as an antimicrobial agent by inhibiting bacterial survival but also as an anti-gastritis agent by reducing virulence and colonization mechanisms.

The drug-likeness and oral bioavailability of chamomile compounds were evaluated using Lipinski’s Rule of Five, followed by an analysis of their ADMET properties. This method offers insights into how these compounds can cross physiological barriers, including the gastrointestinal tract and the blood-brain barrier. It also predicts bioavailability based on interactions with drug-metabolizing enzymes such as cytochrome P450.

Myo-inositol, stearic acid, palmitic acid, cis-11,14-eicosadienoic acid, oleic acid, and 9,12-octadecadienoic acid each violated only one Lipinski rule, primarily due to high lipophilicity (Log *P* > 5). Xylonic acid and benzoic acid did not violate any Lipinski rules. None of the tested compounds inhibited key microsomal cytochromes (CYP1A2, CYP2C19, CYP2C9, CYP2D6, and CYP3A4), suggesting they are unlikely to interfere with the metabolism of co-administered drugs.

Furthermore, none of the compounds were substrates for P-glycoprotein (P-gp), an efflux transporter, which may enhance intracellular bioavailability and therapeutic efficacy. This also helps overcome a potential resistance mechanism, as P-gp has been reported to contribute to *H. pylori* antibiotic resistance [96]. Additionally, all tested compounds were free of Pan Assay Interference Structures (PAINS), indicating reliable biological activity Table 13.

Overall, these findings suggest that chamomile extract is a promising multi-target inhibitor for *H. pylori*, characterized by a favorable safety profile and pharmacokinetic properties. Its synergistic effect with antibiotics may further improve the efficacy of current treatments. Although results with human-derived *H. pylori* isolates are promising, additional in vivo studies and clinical trials are necessary to fully evaluate the safety and therapeutic potential of chamomile extract.

## Conclusion

This study showed that chamomile ethanolic extract has significant in vitro inhibitory activity against *H. pylori*, with varying effectiveness among different isolate groups. It also demonstrated a positive synergistic effect when combined with tested antibiotics. These findings highlight the potential of chamomile extract as a new therapeutic or preventive agent against *H. pylori*, providing a possible approach to combat the growing issue of antibiotic resistance. Further research, including comprehensive in vitro and in vivo toxicity assessments, is needed to evaluate safety and confirm the extract’s effectiveness in vivo.

## Supplementary Information

Supplementary Material 1.

## Data Availability

No datasets were generated or analysed during the current study.

## Abbreviations

ADC: Aspartate α-decarboxylase enzyme
ADMET: Absorption, Distribution, Metabolism, Excretion, and Toxicity
AX: Amoxicillin
BabA: Blood group antigen-binding adhesin
BBB: Blood-brain barrier
BSTFA: Bis(trimethylsilyl) trifluoroacetamide
DS visualizer: Discovery Studio Visualizer
CagA: Cytotoxin-associated gene A
CLR: Clarithromycin
CLSI: Clinical and Laboratory Standards Institute
EUCAST: European Committee on Antimicrobial Susceptibility Testing
FA: Fructose-1,6-bisphosphate aldolase
FTIR: Fourier Transform Infrared Spectroscopy
GCH II: GTP cyclohydrolase II
GC-MS: Gas Chromatography-Mass Spectrometry
GIA: Gastrointestinal absorption
GIT: Gastrointestinal tract
LEV: Levofloxacin
LPP20: Lipopolysaccharide-binding protein 20
LuxS: S-ribosylhomocysteinase
MALT lymphoma: Mucosa-associated lymphoid tissue lymphoma
MBC: Minimum bactericidal concentration
MET: Metronidazole
MIC: Minimum inhibitory concentration
MMFF94: Merck Molecular Force Field 94
PAINS: Pan Assay Interference
PBS: Phosphate buffer saline
PDB: Protein data bank
PDBQT: Protein data bank charges and torsions
PgP: P-glycoprotein transporter
PPI: Proton pump inhibitor
RA: Rifampicin
RUT: Rapid urease test
SabA: Sialic acid-binding adhesion
SDF: Structure Data File
TE: Tetracycline
TMCS: Tri-methyl chlorosilane
VacA: Vacuolating cytotoxin A
