# Supplementary material for: Synergistic potentiation of antibiotics by chamomile phytochemicals against multidrug-resistant Helicobacter pylori
Source: Gut Pathog. 2025 Dec 15;17:105. doi: 10.1186/s13099-025-00777-2 (PMC12703921; doi:10.1186/s13099-025-00777-2)
Supplement: Supplementary file 1 — Supplementary Material 1. [file 13099_2025_777_MOESM1_ESM.docx]

**Table S1.** Minimum inhibitory concentration (MIC) and minimum bactericidal concentration of chamomile extract (mg/mL) against *H. pylori* isolates.

| **isolate number** | **MIC of chamomile extract (mg/mL)** | **MBC of chamomile extract (mg/mL)** |
| --- | --- | --- |
| **1** | 3.12 | 3.12 |
| **2** | 1.56 | 3.12 |
| **3** | 3.12 | 3.12 |
| **4** | 3.12 | 3.12 |
| **5** | 3.12 | 3.12 |
| **6** | 3.12 | 3.12 |
| **7** | 1.56 | 3.12 |
| **8** | 3.12 | 3.12 |
| **9** | 3.12 | 3.12 |
| **10** | 1.56 | 3.12 |
| **11** | 6.25 | 12.5 |
| **12** | 3.12 | 3.12 |
| **13** | 6.25 | 12.5 |
| **14** | 3.12 | 3.12 |
| **15** | 1.56 | 3.12 |
| **16** | 6.25 | 12.5 |
| **17** | 3.12 | 3.12 |
| **18** | 3.12 | 3.12 |
| **19** | 6.25 | 12.5 |
| **20** | 3.12 | 3.12 |
| **mean** | 3.43 | 4.99 |

**Table S2.** Represent the different interactions between control ligands and target proteins of *H. pylori*.

| **Target within**  ***H. pylori*** | **Control Ligand** | **Types of interactions** | **Bond length (Å)** |
| --- | --- | --- | --- |
| **Urease** | **Acetohydroxamic Acid** | **Hydrogen bonds with ASP B:362, ALA B:365, HIS B: 221 and HIS B: 274**  **Metal- acceptor interactions: NI B:3001 and NI B:3002**  **Unfavorable interaction with GLY B:279**  **Van der waals interaction with ALA B:169, HIS B: 136, HIS B: 248, MET B: 366 and THR B:300** | **For H- bond: (2.05-2.93Å)**  **Metal-acceptor: (2.53-3.27 Å)**  **Unfavorable interaction: 2.70 Å** |
| **Lipoprotein 20 (LPP20; HP 1456)** | **Mitomycin (control drug)** | **Hydrogen bonds with ASN A: 96,**  **π - π stacked interaction with PHE A:65**  **π -Alkyl interaction with ALA A:56**  **Van der waals interaction with GLU A: 53, GLY A: 67, ASN A: 92, LEU A: 52 and LEU A: 66** | **For H-bond:(2.25- 2.96Å)**  **π - Alkyl interaction with 5.03 Å**  **π-π interaction with 2.96** **Å** |
| **Aspartate α-decarboxylase (ADC) enzyme** | **Aspartate** | **Hydrogen bonds with THR A:57 and ASN A:71**  **Van der waals interaction with TYR A:58, GLY A:72, ALA A:73, ALA A:74, ILE A: 60 and TYR B:22**  **Unfavorable interactions : GLY B:27, ILE A:26 and ASN A:71**  **Charge –charge interaction with ILE A:26** | **For H-bond:(2.11-2.78** **Å)**  **Charge –charge interaction : 5.09** **Å** |
| **Blood antigen binding adhesion (BabA)** | **Lewis b antigen** | **Hydrogen bonds with SER A:199, CYS A:189, GLY A:191, ASP A:192, ASN A:194, GLN A:207 and ASP A:233**  **Carbon hydrogen bonds with SER A:190 and TYR A:245**  **Alkyl interaction with VAL A:231**  **Van der waals interaction with ASN A:206, LYS A:208, SER A:234, SER A:244, THR A:246, GLN A:200 and SER A:198**  **Unfavorable interaction with GLY A:191 and GLY A: 193** | **For H-bond:(1.99-2.82** **Å)**  **For C-H bond:(3.27-3.59** **Å)**  **For alkyl interaction: 3.46** **Å**  **Unfavorable interaction : (2.20-2.79** **Å)** |
| **fructose-1,6-bisphosphate aldolase (FBA)** | **3-(hydroxy[(phosphonooxy) acetyl]amino}propyl dihydrogen phosphate (PH4)** | **Hydrogen bonds with ASP A:82, GLY A:181, HIS A:210, ASN A:253, ASP A:255, THR A:256 and ARG B:280**  **Carbon hydrogen bonds with HIS A:180, GLY A:211 and ASP A:255**  **π-donor interaction with HIS A:180**  **salt bridge with LYS A:184**  **charge- charge interaction with LYS A:184, ARG B: 280 and ARG A : 259**  **Metal-acceptor interaction with ZN A:308**  **Van dar waals interaction with GLY A:51, THR A:254, SER A:213, ASP A:257, ALA A:212, SER A:179, GLN A: 47, HIS A: 83** | **For H-bonds:(1.90-3.35** **Å)**  **For C-H bonds:(3.44-3.75** **Å)**  **For π-donor interaction:3.70** **Å**  **For salt bridge:1.98** **Å**  **Charge- charge interaction : (3.15-4.91** **Å)**  **Metal-acceptor interaction : 2.20** **Å** |

**Fig. S1.** Shows a 2D representation of interactions between control ligands and active site residues of the target protein. A. represents the interaction of Acetohydroxamic Acid (control ligand) with urease. B. represents the interaction of Lewis B antigen (control ligand) with BabA protein. C. represents the interaction of Mitomycin (control drug) with LPP20 (HP 1456). D. represents the interaction of aspartate (control ligand) with Aspartate α-decarboxylase (ADC) enzyme. E. represents the interaction of 3-(hydroxy[(phosphonooxy)acetyl]amino)propyl dihydrogen phosphate (PH4) (control ligand) with fructose-1,6-bisphosphate aldolase.

| A | B |
| --- | --- |
| 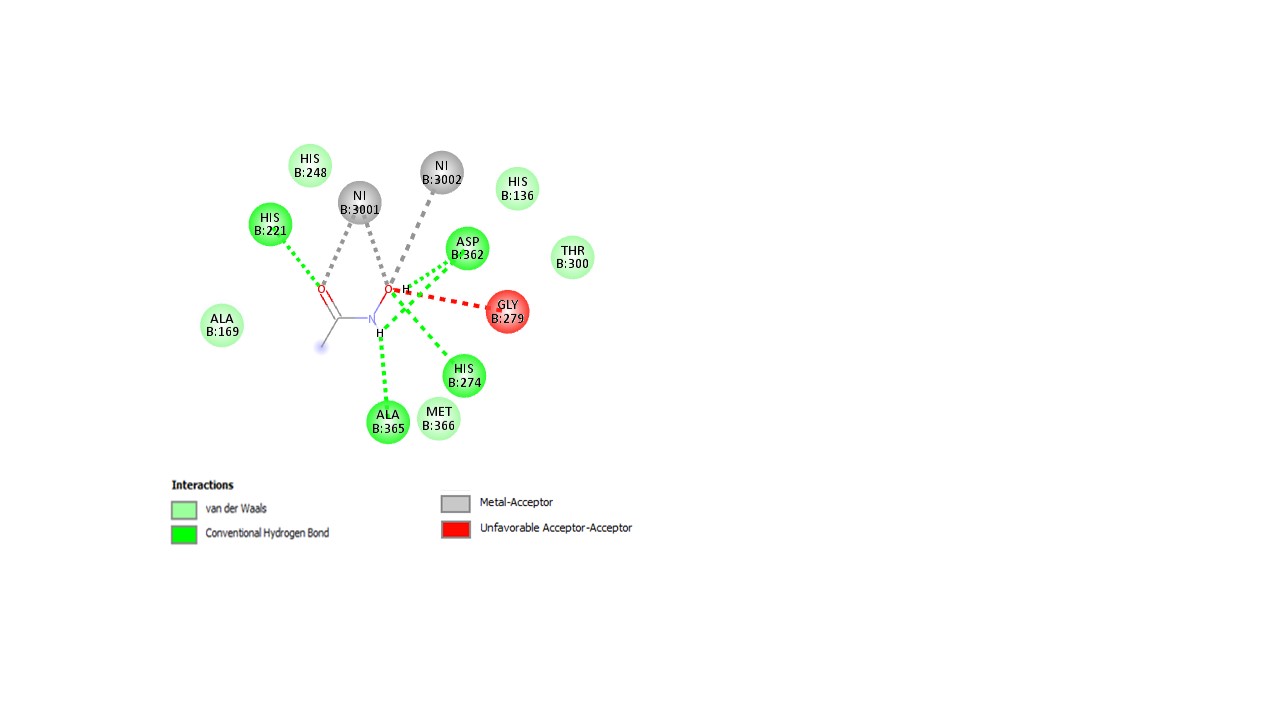 | 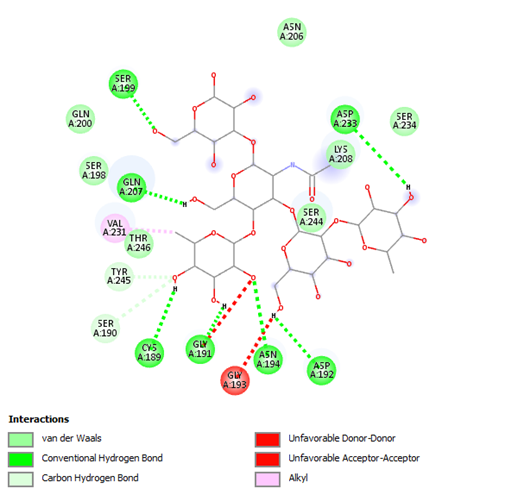 |
| C | D |
| 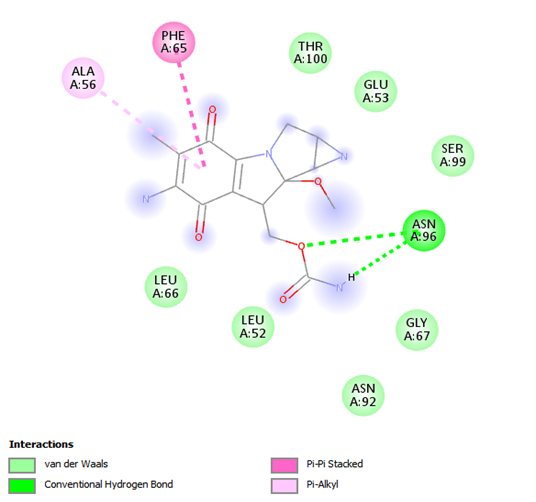 | 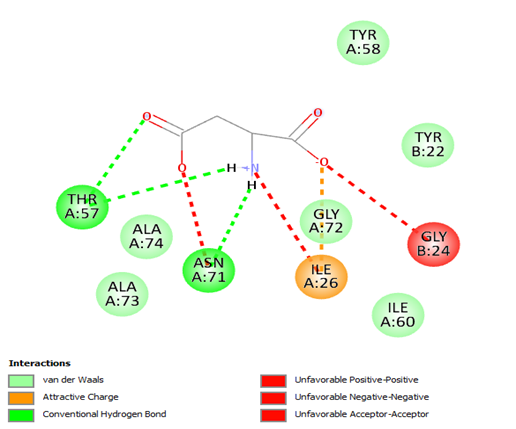 |
| E | |
| 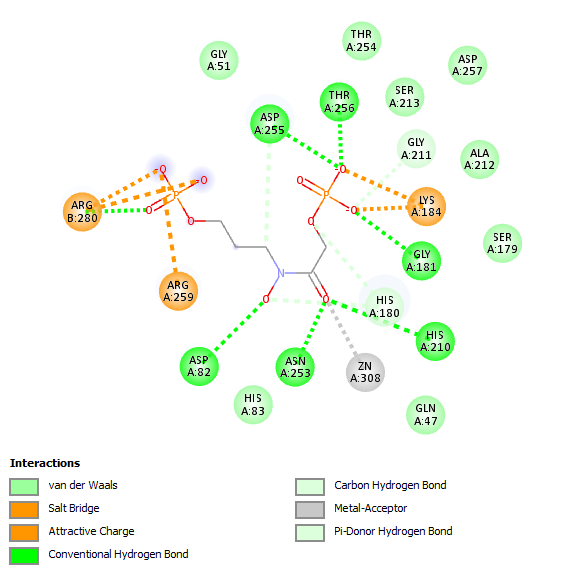 | |
